# Critical Role of Zur and SmtB in Zinc Homeostasis of Mycobacterium smegmatis

**DOI:** 10.1128/mSystems.00880-19

**Published:** 2020-04-21

**Authors:** Elke Goethe, Kristin Laarmann, Janita Lührs, Michael Jarek, Jochen Meens, Astrid Lewin, Ralph Goethe

**Affiliations:** a Institute for Microbiology, Department of Infectious Diseases, University of Veterinary Medicine Hannover, Hannover, Germany; b Genome Analytics, Helmholtz Centre for Infection Research, Braunschweig, Germany; c FG16 Mycotic and Parasitic Agents and Mycobacteria, Robert Koch Institute, Berlin, Germany; University of Wisconsin−Madison

**Keywords:** zinc homeostasis, zinc transporter, zinc starvation, zinc excess, Zur regulon, SmtB regulon, zinc regulation, *zitA*, zinc import, zinc export, transcriptomics, mycobacteria, *znuABC*, alternative ribosomal proteins, chromatin immunoprecipitation, coregulation, export, import

## Abstract

Zinc homeostasis is crucial for bacterial cells, since imbalances affect viability. However, in mycobacteria, knowledge of zinc metabolism is incomplete. Mycobacterium smegmatis (MSMEG) is an environmental, nonpathogenic *Mycobacterium* that is widely used as a model organism to study mycobacterial metabolism and pathogenicity. How MSMEG maintains zinc homeostasis is largely unknown. SmtB and Zur are important regulators of bacterial zinc metabolism. In mycobacteria, these regulators are encoded by an operon, whereas in other bacterial species, SmtB and Zur are encoded on separate loci. Here, we show that the *smtB*-*zur* operon is consistently present within the genus *Mycobacterium* but otherwise found only in *Nocardia*, *Saccharothrix*, and Corynebacterium diphtheriae. By RNA deep sequencing, we determined the Zur and SmtB regulons of MSMEG and compared them with transcriptional responses after zinc starvation or excess. We found an exceptional genomic clustering of genes whose expression was strongly induced by *zur* deletion and zinc starvation. These genes encoded zinc importers such as ZnuABC and three additional putative zinc transporters, including the porin MspD, as well as alternative ribosomal proteins. In contrast, only a few genes were affected by deletion of *smtB* and zinc excess. The zinc exporter ZitA was most prominently regulated by SmtB. Moreover, transcriptional analyses in combination with promoter and chromatin immunoprecipitation assays revealed a special regulation of the *smtB*-*zur* operon itself: an apparently zinc-independent, constitutive expression of *smtB*-*zur* resulted from sensitive coregulation by both SmtB and Zur. Overall, our data revealed yet unknown peculiarities of mycobacterial zinc homeostasis.

**IMPORTANCE** Zinc is crucial for many biological processes, as it is an essential cofactor of enzymes and a structural component of regulatory and DNA binding proteins. Hence, all living cells require zinc to maintain constant intracellular levels. However, in excess, zinc is toxic. Therefore, cellular zinc homeostasis needs to be tightly controlled. In bacteria, this is achieved by transcriptional regulators whose activity is mediated via zinc-dependent conformational changes promoting or preventing their binding to DNA. SmtB and Zur are important antagonistically acting bacterial regulators in mycobacteria. They sense changes in zinc concentrations in the femtomolar range and regulate transcription of genes for zinc acquisition, storage, and export. Here, we analyzed the role of SmtB and Zur in zinc homeostasis in Mycobacterium smegmatis. Our results revealed novel insights into the transcriptional processes of zinc homeostasis in mycobacteria and their regulation.

## Abstract (precis)

Zinc is crucial for many biological processes, as it is an essential cofactor of enzymes and a structural component of regulatory and DNA binding proteins. Hence, all living cells require zinc to maintain constant intracellular levels. However, in excess, zinc is toxic. Therefore, cellular zinc homeostasis needs to be tightly controlled. In bacteria, this is achieved by transcriptional regulators whose activity is mediated via zinc-dependent conformational changes promoting or preventing their binding to DNA. SmtB and Zur are important antagonistically acting bacterial regulators in mycobacteria. They sense changes in zinc concentrations in the femtomolar range and regulate transcription of genes for zinc acquisition, storage, and export. Here, we analyzed the role of SmtB and Zur in zinc homeostasis in Mycobacterium smegmatis. Our results revealed novel insights into the transcriptional processes of zinc homeostasis in mycobacteria and their regulation.

## INTRODUCTION

In living eukaryotic and bacterial cells, zinc plays an essential role in numerous cellular processes, such as DNA replication, transcription, translation, DNA binding, and many enzymatic reactions (1). Thus, it is essential for the survival of eukaryotes and bacteria. However, as with iron and copper, the bioavailability of zinc is very low (2). Hence, depending on their environmental niches, bacteria are exposed to various zinc concentrations (3, 4). Zinc (and other trace metals) also play a role in innate immunity. For example, macrophages can actively fend off bacterial infection by increasing or depleting zinc, thereby causing either intoxication or starvation of the bacteria (nutritional immunity) (5). Therefore, in the environment as well as in the host, bacteria need mechanisms to efficiently regulate zinc homeostasis. Zinc homeostasis is achieved by the expression of regulated or nonregulated, specific or nonspecific, energy-dependent or -independent systems. Cation diffusion facilitators (CDFs), such as CzcD of Bacillus subtilis (6) and ZitB of Escherichia coli (7), allow regulated, specific, ATP-independent uptake or efflux (8), whereas the activity of specific low-affinity zinc importers of the ZIP family, e.g., ZupT of E. coli (9), or high-affinity transporters such as P_1_-type ATPases, e.g., ZntA of E. coli (10) and CtpC of Mycobacterium tuberculosis (MTB) (11), is mostly ATP dependent (12, 13). The most prominent family of inducible high-affinity bacterial zinc transporters is the common ATP binding cassette (ABC) importer ZnuABC, which is an important virulence factor in many bacteria, e.g., E. coli, Salmonella enterica, Campylobacter jejuni, and Yersinia pestis (14–19).

Bacteria are able to sense alterations of intracellular zinc concentration in the femtomolar range by zinc-responsive regulators (20). They regulate the expression of zinc uptake, export, or storage systems. Examples include DtxR (diphtheria toxin regulator)-like regulators such as TroR of Treponema pallidum, MerR (metal-responsive regulator)-like regulators such as ZntR of E. coli, and ArsR (arsenite-sensitive regulator)-like repressors such as SmtB of *Synechococcus* species and CzrA of Staphylococcus aureus, and Zur (zinc uptake regulator, also known as FurB) of MTB, a member of the Fur (ferric uptake regulator) family (21–26).

Knowledge of zinc homeostasis and its regulation in mycobacteria is very limited. Most information concerning pathogenic species is based on heterologous studies in the nonpathogenic Mycobacterium smegmatis (MSMEG). These studies indicate that the antagonistically acting repressors SmtB and Zur regulate zinc homeostasis by using zinc as a cofactor (27). Thereby they regulate bacterial responses to changing intracellular zinc availability by activating or repressing the expression of importer or exporter genes. During starvation, apo-Zur is released from the DNA, allowing zinc import by derepression of zinc importer genes such as MTB *znuABC* and *yciC* (28). Similar activity of Zur was observed in the ruminant pathogen Mycobacterium avium subsp. *paratuberculosis* (MAP) (29). Holo-SmtB detaches from the DNA when zinc is in excess, thereby allowing gene transcription of zinc exporters such as ZitA of MTB (30). Zinc-dependent regulation of *smtB* expression has been demonstrated for MTB and MAP (29, 31). In these species, SmtB has been shown to be cotranscribed with Zur from an operon (here referred to as *smtB*-*zur*) which seems to be autoregulated by SmtB (28). Cotranscription and autoregulation has also been proposed for MSMEG (30), suggesting a general regulation concept of the operon in mycobacteria.

Little is known about the influence of zinc on gene expression in mycobacteria. The regulons of MTB Zur and the zinc starvation regulon of MAP have been identified (28, 29). These studies revealed a Zur-dependent and zinc starvation-dependent induction of a set of genes, which encode alternative ribosomal proteins (ARPs). ARPs, in contrast to their zinc-containing paralogues, do not use zinc as a structural component. In addition, the virulence-associated mycobacterial type VII secretion system ESX-3, the zinc importer *znuABC*, and CobW-like chaperones were all induced upon Zur deletion or zinc starvation (28, 29, 32, 33). However, direct involvement of Zur in regulation was based on binding site predictions only and was not experimentally confirmed (28, 29). Knowledge of the role of SmtB in zinc homeostasis in mycobacteria is limited to prediction of its binding site in MTB and studies of its promoter in MSMEG (30).

In nonpathogenic mycobacteria, e.g., MSMEG, very few studies have been published on zinc homeostasis. These include the presence of a *zitA* homologue (34), the zinc-dependent DNA repair protein KU, which is important for zinc resistance (35), and the involvement of mycobacterial protein Y (MPY) in the hibernation of alternative ribosomes (36).

In the present study, we applied a comprehensive transcriptional approach to investigate the *smtB*-*zur* operon of mycobacteria in the context of MSMEG responses to zinc starvation and excess as well as the regulons of SmtB and Zur. Our results provide novel insights into zinc-dependent gene regulation in MSMEG and reveal additional putative zinc transport systems. Moreover, we demonstrated the relevance of SmtB and Zur during zinc stress and starvation and characterized details of their own regulation. The results indicate that the regulation of zinc homeostasis in MSMEG is more complex than that in pathogenic mycobacteria. Overall, we provide novel findings which contribute to our understanding of mycobacterial responses to changing zinc concentrations.

## RESULTS

### The *smtB*-*zur* operon structure is conserved in mycobacteria.

**FIG 1:**
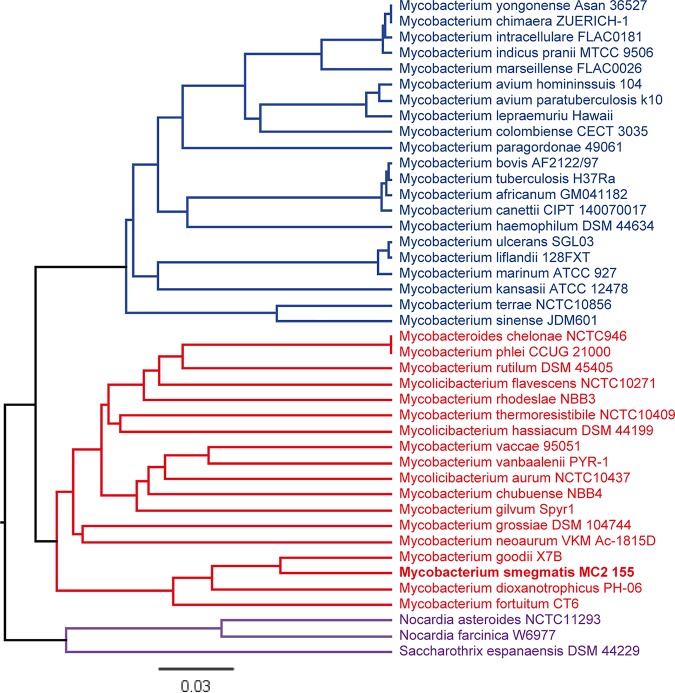
Phylogenetic analysis of *smtB*-*zur* operon organization. Sequences homologous to MSMEG *smtB*-*zur* (MSMEG_4486-87; accession no. NC_008596; bases 4569200 to 4569951) were identified by BLASTN. A distance tree of results (neighbor joining tree, maximum sequence difference of 0.75) was generated and displayed using Geneious 11.1.5. Closely related clusters are indicated by color.

Regulation of zinc homeostasis in mycobacteria is achieved by the antagonistic activity of the regulators SmtB and Zur (27). These regulators are also present in other bacterial genera, but, in contrast to mycobacteria, they are encoded on separate genes which are not organized in an operon. Hence, we were interested in whether this organization is specific for mycobacteria. We performed phylogenetic analyses based on the sequence of *smtB*-*zur* of MSMEG in NCBI BLASTN and subsequent neighborhood joining analysis. Of the 42 strains harboring the *smtB*-*zur* operon, 39 were mycobacterial strains. They could be grouped into mainly environmental/nonpathogenic and mainly pathogenic species. The remaining three strains were genera of the phylum *Actinobacteria*, e.g., *Nocardia* species and *Saccharothrix* species (Fig. 1). Furthermore, an operon was also found in Corynebacterium diphtheriae but with significantly lower sequence homology (37). Thus, the arrangement of *smtB*-*zur* on an operon as present in mycobacteria is otherwise observed only in a very small group of actinobacteria. Operon structure was confirmed in selected species by extracting the genome regions homologous to MSMEG *zur* and *smtB* and by performing multiple sequence alignments. The coding sequences homologous to *zur* and *smtB* are overlapping in MSMEG, Mycobacterium thermoresistibile, MTB, and Saccharothrix espanaensis. In Nocardia asteroides, the two coding sequences are separated by 4 bp (see Fig. S2 in the supplemental material).

### Zur and SmtB contribute to zinc resistance in MSMEG.

To get more insight into the role of SmtB and Zur in zinc homeostasis of MSMEG, we determined their regulons. For this, we used the previously published regulator mutant MSMEGΔ*zur* (29) and generated the deletion mutants MSMEGΔ*smtB* and MSMEGΔs*mtB*Δ*zur* as well as the complemented strains MSMEGΔ*smtB*^C^, MSMEGΔ*zur*^C^, and MSMEGΔ*smtB*^C^Δ*zur*^C^.

Mutation, complementation, and polar effects on the adjacent gene MSMEG_4488 were controlled by quantitative real-time PCR (qRT-PCR) (Fig. S3). As expected, *smtB* expression was abolished in MSMEGΔ*smtB* and MSMEGΔ*smtB*Δ*zur* but still present in MSMEGΔ*zur*. Vice versa, expression of *zur* was observed in MSMEGΔ*smtB* but was not detectable in MSMEGΔ*zur* or MSMEGΔ*smtB*Δ*zur*. Interestingly, expression of *smtB* was higher in MSMEGΔ*zur* and expression of *zur* was higher in MSMEGΔs*mtB* than in the wild type, suggesting that both regulators contribute to their own expression. Complementation restored the wild-type phenotype; mRNA expression of the respective genes on the integrated complementation vector was higher in the complemented strains than in the wild type. The adjacent gene, MSMEG_4488, was expressed in all mutated strains (Fig. S3).

**FIG 2:**
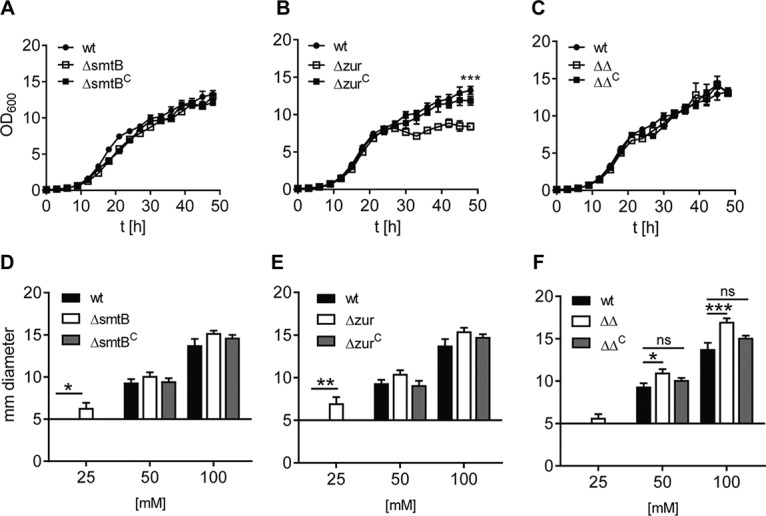
Growth behavior of MSMEG deletion mutants (A to C) and impact of Zur and SmtB on MSMEG zinc tolerance (D to F). (A to C) Growth curve. MSMEGwt (filled circles), MSMEGΔ*smtB* (A), MSMEGΔ*zur* (B), and MSMEGΔ*smtB*Δ*zur* (C) mutants (open squares) and the complemented strains (filled squares) were grown in MB. Four growth experiments were performed in duplicate. Statistical analysis for time point 48 h was performed using one-way analysis of variance (ANOVA) (Kruskal Wallis) with a *P* of <0.0005 (***). (D to F) ZoI assay. MSMEGwt (black bars) and the MSMEGΔ*smtB* (D), MSMEGΔ*zur* (E), and MSMEGΔ*smtB*Δ*zur* (F) mutants (white bars) and complemented strains (gray bars) were spread on LB agar. ZnSO_4_ was applied to filter discs (diameter, 5 mm) at final concentrations of 25, 50, and 100 mM. Shown are the results of three independent replicates (in triplicate). Statistical analysis was performed using one-way ANOVA (Kruskal-Wallis) with *P* values of <0.05 (*), <0.005 (**), and <0.0005 (***). ns, nonsignificant.

Growth kinetics of all strains were monitored in standard MB (see Materials and Methods). Growth of MSMEGΔ*smtB* and the double mutant MSMEGΔ*smtB*Δ*zur* was comparable to that of the wild type (Fig. 2A and C). Only MSMEGΔ*zur* showed a significantly reduced growth and had entered the stationary phase already after 27 h. Complementation restored the wild-type phenotype (Fig. 2B). To analyze differences in zinc tolerance of the different mutants, we used a plating assay with increasing zinc concentrations. Briefly, strains were spread on agar and ZnSO_4_ was applied to filter discs in increasing concentrations. No growth inhibition was visible with water control. At low ZnSO_4_ concentrations (25 mM), growth of MSMEGΔ*smtB* and MSMEGΔ*zur* was significantly impaired, as indicated by a larger zone of growth inhibition (ZoI), which was not observed for wild-type MSMEG (MSMEGwt) and the complemented strains (Fig. 2D and E). The double mutant showed significantly reduced growth at higher concentrations (50 or 100 mM) (Fig. 2F). Hence, all mutants were affected in growth, most likely by impaired abilities to maintain zinc homeostasis.

### Responses of MSMEG to changes in zinc homeostasis.

The above-described data suggest an involvement of SmtB and Zur in control of zinc homeostasis. To further dissect the underlying molecular mechanisms, we analyzed the transcriptional response of MSMEGwt cultures treated with the zinc chelator TPEN [*N*,*N*,*N′*,*N′*-tetrakis (2-pyridylmethyl) ethylenediamine] (10 μM) or exposed to ZnSO_4_ (500 μM). Gene expression was determined by RNA deep sequencing (RNA-Seq), and the transcriptome of MSMEG treated with TPEN or exposed to ZnSO_4_ was compared to that of the untreated control.

**FIG 3:**
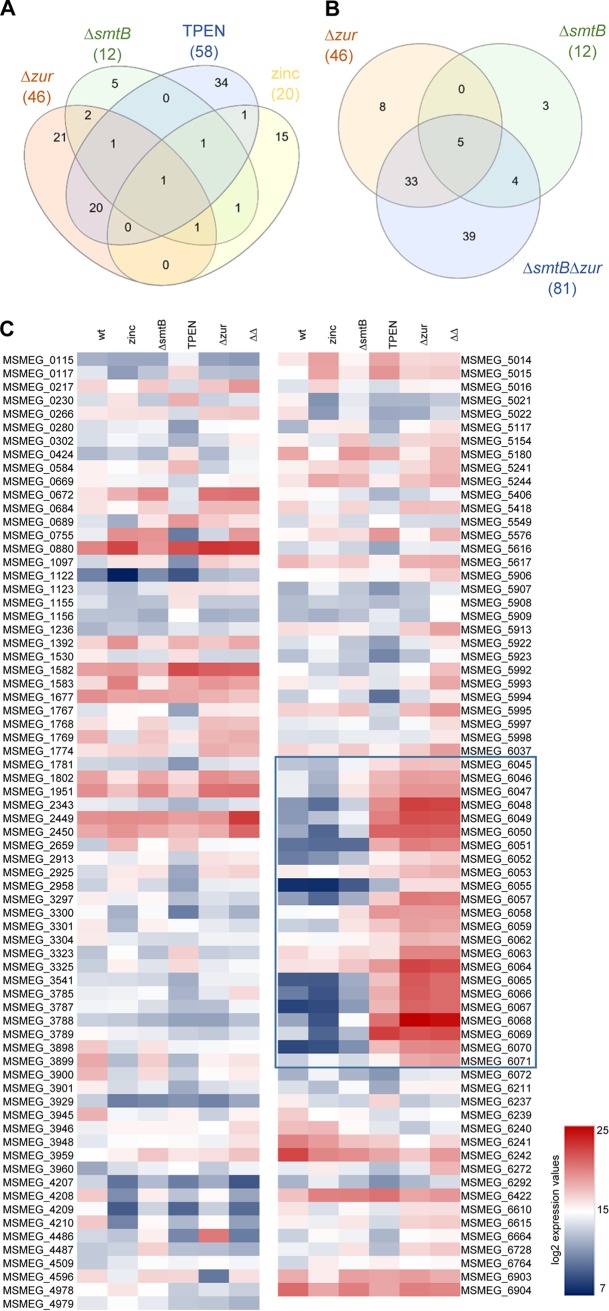
Congruencies of expression of MSMEG zinc-dependent genes. Venn diagrams of differentially expressed genes from RNA-Seq. (A) MSMEGΔ*smtB*, MSMEGΔ*zur*, and MSMEGwt treated with TPEN or zinc; (B) MSMEGΔ*smtB*, MSMEGΔ*zur*, and MSMEGΔ*smtB*Δ*zur.* (C) Heat map of differentially expressed genes. Shown are the average normalized expression values of three independent replicates of MSMEGwt, untreated or treated with TPEN or zinc, MSMEGΔ*smtB*, MSMEGΔ*zur*, and MSMEGΔ*smtB*Δ*zur* (ΔΔ) obtained from RNA-Seq and presented as log_2_. The highly induced gene cluster MSMEG_6045-6071 in MSMEGwt with TPEN, MSMEGΔ*zur*, and MSMEGΔ*smtB*Δ*zur* is highlighted by a blue box.

Zinc starvation upon TPEN treatment resulted in the differential expression of 58 genes (Fig. 3A; Table 1), which are organized in 10 operons (as predicted by Rockhopper analysis). Higher expression was observed for 26 genes and lower expression for 32 genes. More than 60% of the differentially expressed genes exhibited 4- to 7-fold changes. These genes encode proteins involved in energy conversion (MSMEG_1768, MSMEG_3541), transport (MSMEG_2925), gene regulation (MSMEG_1769), and stress and immune responses (MSMEG_3945, MSMEG_5617). Others are involved in metabolic and enzymatic processes (MSMEG_0115, MSMEG_0117, MSMEG_0266, MSMEG_0280, MSMEG_0684, MSMEG_1097, MSMEG_1155, MSMEG_1156, MSMEG_2343, MSMEG_2913, MSMEG_3304, MSMEG_3785, MSMEG_3929, MSMEG_5616, MSMEG_6071, MSMEG_6664) or are of unknown function (MSMEG_0669, MSMEG_0672, MSMEG_1767, MSMEG_1774, MSMEG_1781, MSMEG_1802, MSMEG_1951, MSMEG_2958, MSMEG_5154, MSMEG_6211, MSMEG_6610, MSMEG_6615, MSMEG_6728).

**TABLE 1:** MSMEG genes affected by zinc starvation after TPEN treatment

RCN^*a*^	Annotation^*c*^	Orthologous gene (% similarity)		*q* value^*d*^	Fold change^*e*^	Putative function^*f*^
		MTB	MAP			
*dehII*	*MSMEG_0115*	—	—	<0.0001	6.22	Haloacid dehalogenase
—^*b*^	*MSMEG_0117*	Rv2296 (44.1)	MAP2057 (43.5)	<0.0001	5.61	Hydrolase
—	MSMEG_0266	—	MAP2144 (77.9)	<0.0001	−5.08	Arginine decarboxylase
—	MSMEG_0280	Rv0217c (65.2)	MAP3655c (64.2)	<0.0001	−6.0	Alpha/beta-hydrolase
—	MSMEG_0669	—	—	<0.0001	−4.73	Hypothetical protein
—	MSMEG_0672	—	—	<0.0001	−4.29	Hypothetical protein
—	MSMEG_0684	—	—	<0.0001	−4.91	Aldehyde oxidase
—	MSMEG_0755	Rv0359 (68.8)	MAP3865c (76.8)	<0.0001	−34.0	Cobalt-zinc-cadmium resistance protein
—	MSMEG_1097	—	—	<0.0001	−6.5	Glycosyl transferase family protein
—	MSMEG_1123	—	MAP1730c (43.1)	<0.0001	7.56	Cobalamin synthesis protein
—	*MSMEG_1155*	—	—	<0.0001	4.63	Carnitinyl-CoA dehydratase
—	*MSMEG_1156*	—	—	<0.0001	5.0	Dihydrodipicolinate synthetase
—	*MSMEG_1767*	—	MAP3409c (42.5)	<0.0001	−5.64	Hypothetical protein
—	*MSMEG_1768*	—	—	<0.0001	−4.62	Flavodoxin
—	*MSMEG_1769*	—	MAP0383 (43.7)	<0.0001	−4.04	UsfY protein
—	MSMEG_1774	—	—	<0.0001	−4.04	Hypothetical protein
—	MSMEG_1781	—	—	<0.0001	−5.43	Hypothetical protein
—	MSMEG_1802	—	MAP2952c (74.6)	<0.0001	−4.28	ChaB protein
—	MSMEG_1951	—	—	0.00095	−4.14	Translation initiation factor, IF2 family protein
—	MSMEG_2343	—	MAP3076 (70.0)	<0.0001	−4.43	Methylesterase
—	MSMEG_2913	—	—	<0.0001	−4.53	Hydrolase
—	MSMEG_2925	—	—	<0.0001	−4.17	Permease membrane component
—	MSMEG_2958	—	MAP1039 (54.2)	<0.0001	−4.2	Hypothetical protein
—	MSMEG_3304	—	—	<0.0001	−4.79	Succinate semialdehyde dehydrogenase
—	MSMEG_3541	Rv2877c (65.5)	MAP2941c (60.8)	<0.0001	−4.17	Cytochrome *c* biogenesis protein transmembrane region
—	MSMEG_3785	—	—	<0.0001	−4.71	PfkB family protein carbohydrate kinase
—	MSMEG_3929	—	—	<0.0001	−6.0	[NiFe] hydrogenase subunit delta
—	MSMEG_3945	Rv2005c (47.8)	MAP1741c (51.1)	<0.0001	−5.20	Universal stress protein family protein
—	MSMEG_5154	—	MAP2627c (76.6)	<0.0001	−4.71	Hypothetical protein
—	MSMEG_5406	—	—	<0.0001	−8.97	Hypothetical protein
—	*MSMEG_5616*	Rv0911 (61.3)	—	<0.0001	−4.0	Glyoxalase/bleomycin resistance protein/dioxygenase
—	*MSMEG_5617*	—	—	<0.0001	−4.45	Immunogenic protein MPT63
—	*MSMEG_6045*	Rv2060 (43.7)	MAP3774c (70.1)	<0.0001	21.29	Heavy metal ABC transporter inner membrane protein
—	*MSMEG_6046*	—	MAP3775c (61.2)	<0.0001	19.04	ABC transporter ATP-binding protein
—	*MSMEG_6047*	—	MAP3776c (56.4)	<0.0001	43.8	Cation ABC transporter periplasmic cation-binding protein
—	*MSMEG_6048*	—	MAP3772c (72.6)	<0.0001	456.0	Cobalamin synthesis protein/P47K
—	*MSMEG_6049*	—	—	<0.0001	624.5	Secreted protein
—	*MSMEG_6050*	—	—	<0.0001	556.5	ABC-type Zn uptake system solute-binding lipoprotein
—	*MSMEG_6051*	—	—	<0.0001	227.0	Pseudo-ABC transporter trans-membrane protein
—	*MSMEG_6052*	—	—	<0.0001	54.0	ABC transporter ATP-binding protein
—	MSMEG_6055	—	—	<0.0001	28.0	Class I SAM-dependent methyl-transferase
—	MSMEG_6057	—	—	<0.0001	52.0	MspD protein
—	*MSMEG_6058*	Rv3270 (77.2)	MAP3384 (77.7)	<0.0001	17.60	Cadmium-transporting P-type ATPase
—	*MSMEG_6059*	Rv3269 (71.6)	MAP3383 (69.1)	<0.0001	15.22	Hypothetical protein
—	MSMEG_6064	—	—	<0.0001	6.52	Lipoprotein
*rpsR*	*MSMEG_6065*	Rv2055c (63.4)	MAP3767c (63.9)	<0.0001	521.0	30S ribosomal protein S18
*rpsN*	*MSMEG_6066*	Rv2056c (72.3)	MAP3768c (75.2)	<0.0001	279.8	30S ribosomal protein S14
*rpmG*	*MSMEG_6067*	Rv2057c (87.0)	MAP3769c (92.6)	<0.0001	1,043.5	50S ribosomal protein L33
*rpmB*	*MSMEG_6068*	Rv2058c (82.1)	—	<0.0001	449.65	50S ribosomal protein L28
—	*MSMEG_6069*	Rv0106 (62.1)	MAP3770 (57.8)	<0.0001	406.31 (4,524.0)	CobW/P47K domain-containing protein, MPY recruitment factor (MRF)
*rpmE2*	*MSMEG_6070*	—	MAP3771 (79.3)	<0.0001	1,233.0	50S ribosomal protein L31
—	*MSMEG_6071*	—	MAP0485c (66.9)	<0.0001	5.2	Metallo-beta-lactamase superfamily protein
—	MSMEG_6211	—	—	<0.0001	−4.11	Hypothetical protein
—	MSMEG_6237	—	—	<0.0001	7.4	Class I SAM-dependent methyl-transferase
—	*MSMEG_6610*	—	—	<0.0001	−4.16	Hypothetical protein
—	*MSMEG_6615*	—	—	<0.0001	−4.36	Dithiol-disulfide isomerase
—	MSMEG_6664	—	—	<0.0001	−4.56	Methylenetetrahydrofolate reductase
—	MSMEG_6728	—	—	<0.0001	−4.0	Cytoplasmic protein

a RCN, reference common name.

b —, no reference common name/orthologous gene.

c Italics indicate genes organized in an operon.

d *q* value of differentially expressed genes in MSMEGwt standard culture versus MSMEGwt TPEN culture calculated by Rockhopper analysis. A *q* value of <0.01 is considered significant.

e Gene expression values of MSMEGwt TPEN culture divided by gene expression values of MSMEGwt standard culture from RNA-Seq. Values in parentheses were obtained from qRT-PCR.

f Putative function based on NCBI blastx analysis or the TB database (http://genome.tbdb.org). CoA, coenzyme A.

Differentially expressed genes with a fold change of ≥7 comprised the genes MSMEG_5406 and MSMEG_6237 (encoding hypothetical proteins) and a special cluster of genes, MSMEG_6045-6071, which was more highly expressed after zinc starvation (Fig. 3C). This cluster comprised genes encoding four putative transporters (MSMEG_6049-6051, MSMEG_6052, MSMEG_6058-6059), including the common ZnuABC transporter (MSMEG_6045-6047), two CobW-like proteins (MSMEG_6048 and MSMEG_6069), the latter representing the recently described mycobacterium-specific protein Y (MPY) recruitment factor (MRF) (36), a set of genes encoding zinc-independent alternative ribosomal proteins (ARPs) RpsR, RpsN, RpmG, RpmB, and RpmE2 (MSMEG_6065, MSMEG_6066, MSMEG_6067, MSMEG_6068, MSMEG_6070), a gene encoding the porin MspD (MSMEG_6057), and two genes of unknown function (MSMEG_6055, MSMEG_6064). In addition, ≥7-fold-lower expression upon TPEN treatment was observed for the cobalt-zinc-cadmium resistance gene MSMEG_0755. The encoded protein belongs to the group of CDF transporters, is closely related to the P_1B_-type ATPase CzcD (34), and is a putative homologue of the zinc exporter ZitA (38).

MSMEG responded to zinc excess by differential expression of only 20 genes, i.e., 13 with higher expression and 7 with lower expression, compared to the untreated control (Table 2). They are predicted to be involved in transcription/regulation (MSMEG_6292, MSMEG_6764, MSMEG_6903), metabolic or enzymatic processes (MSMEG_6664, MSMEG_6904), stress response (MSMEG_1392, MSMEG_5117, MSMEG_6242), and (metal) transport (MSMEG_0755, MSMEG_1530, MSMEG_5014, MSMEG_5418) or are of unknown function (MSMEG_0230, MSMEG_0689, MSMEG_3323, MSMEG_3325, MSMEG_5015, MSMEG_5016, MSMEG_5549, MSMEG_6237). Moreover, the expression of MSMEG_6903 and MSMEG_6904 was reduced upon zinc addition. These genes encode a PadR family transcription regulator and a synthase involved in myo-inositol-1-phosphate (MIP) generation, respectively. The MIP protein has been shown to form a complex with zinc (39). Within the group of genes with higher expression, we found a gene cluster putatively involved in zinc transport (MSMEG_5014-5016) and a proline dehydrogenase (MSMEG_5117). However, the most prominently affected gene of the zinc stress transcriptome was MSMEG_0755, which was strongly induced upon zinc excess and, as shown above, had lower expression upon zinc starvation (Table 1). This supports the hypothesis that MSMEG_0755 is involved in zinc export.

**TABLE 2:** MSMEG genes affected by zinc excess

RCN^*a*^	Annotation^*c*^	Orthologous gene (% similarity)		*q* value^*d*^	Fold change^*e*^	Putative function^*f*^
		MTB	MAP			
—^*b*^	MSMEG_0230	Rv0190 (80.0)	MAP3632 (85.9)	<0.0001	5.29	Hypothetical protein
—	MSMEG_0689	—	—	<0.0001	−4.04	Hypothetical protein
—	MSMEG_0755	Rv0359 (68.8)	MAP3865c	<0.0001	14.36	Cobalt-zinc-cadmium resistance protein
—	MSMEG_1392	—	—	<0.0001	5.37	Alcohol dehydrogenase
—	*MSMEG_1530*	—	—	<0.0001	−4.26	Integral membrane protein
—	*MSMEG_3323*	—	MAP0103c (80.2)	<0.0001	4.80	Hypothetical protein
—	MSMEG_3325	—	MAP0102 (91.0)	<0.0001	5.38	Hypothetical protein
—	*MSMEG_5014*	Rv0969 (52.8)	MAP4284 (63.6)	<0.0001	6.25	Zinc/cadmium/cobalt P-type ATPase
—	*MSMEG_5015*	—	—	<0.0001	10.36	Hypothetical protein
—	*MSMEG_5016*	—	—	<0.0001	6.53	Hypothetical protein
—	*MSMEG_5117*	Rv1188 (68.3)	MAP2592c (71.6)	<0.0001	11.75	Proline dehydrogenase
—	MSMEG_5418	—	—	<0.0001	−4.46	Iron permease
—	MSMEG_5549	Rv0943c (51.0)	MAP0887c (71.9)	<0.0001	4.63	Hypothetical protein
—	MSMEG_6237	—	—	<0.0001	−11.22	Hypothetical protein
—	*MSMEG_6242*	—	—	<0.0001	−4.64	Alcohol dehydrogenase → oxidative stress
—	MSMEG_6292	—	MAP1027c (41.9)	<0.0001	4.53	Transcription elongation factor GreA
—	*MSMEG_6664*	—	—	<0.0001	4.17	Methylenetetrahydrofolate reductase
—	MSMEG_6764	—	MAP0155 (43.2)	<0.0001	4.87	TetR family transcriptional regulator
—	*MSMEG_6903*	Rv0047c (91.9)	MAP0061c (88.8)	<0.0001	−5.50	PadR family transcriptional regulator
—	*MSMEG_6904*	Rv0046c (85.7)	MAP0060c (86.8)	<0.0001	−4.58	Myo-inositol-1-phosphate synthase

a RCN, reference common name.

b —, no reference common name/orthologous gene.

c Italics indicate genes organized in an operon.

d *q* value of differentially expressed genes in MSMEGwt standard culture versus MSMEGwt ZnSO_4_ culture calculated by Rockhopper analysis. A *q* value of <0.01 is considered significant.

e Gene expression values of MSMEGwt ZnSO_4_ culture divided by gene expression values of MSMEGwt standard culture from RNA-Seq.

f Putative function based on NCBI blastx analysis or the TB database.

Overall, the MSMEG response to zinc limitation involved a large number of genes associated with zinc homeostasis. The response to zinc excess, however, was less pronounced, as it caused only moderate changes in gene expression, and most of these genes could not be assigned to zinc homeostasis.

### SmtB and Zur control zinc homeostasis.

To dissect the relevance of MSMEG SmtB and Zur for zinc homeostasis, we determined the regulons of both regulators by RNA sequencing of the respective mutants and the MSMEGΔ*smtB*Δ*zur* double mutant. Differentially expressed genes were compared with those of zinc starvation and stress regulons to determine the zinc dependency of SmtB/Zur-regulated genes.

Transcriptome analysis of the MSMEGΔ*zur* mutant revealed 46 differentially expressed genes compared to MSMEGwt. Differential expression of 25 of these genes was congruent with the TPEN or zinc stress transcriptome (Fig. 3A and C). They were classified as Zur and zinc dependently regulated genes (Table 3). The remaining 21 genes were considered Zur dependent but zinc independently regulated (Table 4).

**TABLE 3:** Genes differentially expressed in MSMEGΔ*zur*, zinc dependent (including TPEN, zinc, and Δ*smtB*Δ*zur* results)

RCN^*a*^	Annotation^*c*^	Orthologous gene (% similarity)		*q* value^*d*^	Fold change^*e*^				Putative function^*f*^
		MTB	MAP		Δ*zur*	TPEN	Zn	ΔΔ	
—^*b*^	*MSMEG_1123	—	MAP1730c (43.1)	<0.0001	8.64	7.56		7.50	Cobalamin synthesis protein
—	*MSMEG_6045*	Rv2060 (43.7)	MAP3774c (70.1)	<0.0001	24.62	21.29		24.42	Heavy metal ABC transporter inner membrane protein
—	*MSMEG_6046*	—	MAP3775c (61.2)	<0.0001	20.65	19.04		19.90	ABC transporter ATP-binding protein
—	**MSMEG_6047	—	MAP3776c (56.4)	<0.0001	26.24	43.8		25.50	Cation ABC transporter periplasmic cation-binding protein
—	**MSMEG_6048	—	MAP3772c (72.6)	1	3288.50	456.0		2,903.00	Cobalamin synthesis protein/P47K
—	*MSMEG_6049*	—	—	<0.0001	494.44	624.5		443.56	Secreted protein
—	*MSMEG_6050*	—	—	<0.0001	917.25	556.5		849.0	ABC-type Zn uptake system solute-binding lipoprotein
—	*MSMEG_6051*	—	—	<0.0001	777.0	227.0		632.0	Pseudo ABC transporter transmembrane protein
—	*MSMEG_6052	—	—	<0.0001	18.17	54.00		23.42	ABC transporter ATP-binding protein
—	**MSMEG_6055	—	—	1.00	498.00	28.00		511.00	Class I SAM-dependent methyltransferase
—	MSMEG_6057	—	—	<0.0001	435.00	52.00		393.0	MspD protein
—	*MSMEG_6058*	Rv3270 (77.2)	MAP3384 (77.7)	<0.0001	9.21	17.59		9.59	Cadmium transporting P-type ATPase
—	*MSMEG_6059*	Rv3269 (71.6)	MAP3383 (69.1)	<0.0001	13.42	15.22		11.73	Hypothetical protein
—	MSMEG_6064	—	—	<0.0001	27.42	6.52		21.76	Lipoprotein
*rpsR*	*MSMEG_6065*	Rv2055c (63.4)	MAP3767c (63.9)	1.00	5107.67	521.00		3,080.0	30S ribosomal protein S18
*rpsN*	*MSMEG_6066*	Rv2056c (72.3)	MAP3768c (75.2)	1.00	5778.50	279.80		3,983.5	30S ribosomal protein S14
*rpmG*	*MSMEG_6067*	Rv2057c (87.0)	MAP3769c (92.6)	1.00	5856.67	1043.50		5,015.0	50S ribosomal protein L33
*rpmB*	*MSMEG_6068	Rv2058c (82.1)	—	1.00	5372.69	449.65		4,055.32	50S ribosomal protein L28
—	*MSMEG_6069	Rv0106 (62.1)	MAP3770 (57.8)	1.00	3492.00	406.31		4,340.00	CobW/P47K domain-containing protein
*rpmE2*	*MSMEG_6070*	—	MAP3771 (79.3)	1.00	1460.33	1233.00		1,603.0	50S ribosomal protein L31
—	*MSMEG_6071*	Rv3577 (68.3)	MAP0485c (66.9)	<0.0001	27.95	5.20		33.42	Metallo-beta-lactamase superfamily protein
—	MSMEG_6237	—	—	<0.0001	−6.93	7.40	−11.22	−4.62	Class I SAM-dependent methyltransferase
—	*MSMEG_6240*	—	—	<0.0001	−24.38		(−3.01)	−8.48	Hypothetical protein
—	*MSMEG_6241*	Rv2426c (45.9)	MAP2246c (44.9)	<0.0001	−24.63		(−3.74)	−8.66	ATPase AAA
—	*MSMEG_6242*	—	—	<0.0001	−27.59		−4.64	−8.35	Alcohol dehydrogenase

a RCN, reference common name.

b —, no reference common name/orthologous gene.

c Italics indicate genes organized in an operon. Asterisks indicate the presence of one or more predicted Zur binding sites.

d *q* value of differentially expressed genes in MSMEGwt versus MSMEGΔ*zur* calculated by Rockhopper analysis. A *q* value of <0.01 is considered significant.

e Gene expression values of MSMEGΔ*zur* (Δ*zur*) and MSMEGΔ*smt*BΔ*zur* (ΔΔ) divided by gene expression values of MSMEGwt from RNA-Seq as well as results from transcriptome analysis after TPEN and ZnSO_4_ treatment. Values in parentheses indicate the differential expression of <4 genes which belong to the same operon.

f Putative function based on NCBI blastx analysis or the TB database.

**TABLE 4:** Genes differentially expressed in MSMEGΔ*zur*, zinc independent

RCN^*a*^	Annotation^*c*^	Orthologous gene (% similarity)		*q* value^*d*^	Fold change^*e*^				Putative function^*f*^
		MTB	MAP		Δ*zur*	TPEN	Zn	ΔΔ	
—^*b*^	MSMEG_0584	—	—	<0.0001	−5.30				CoA transferase
*groEL*	MSMEG_0880	Rv0440 (93.4)	MAP3936 (95.0)	<0.0001	7.94			6.62	Molecular chaperone GroEL
—	*MSMEG_1122	—	—	<0.0001	4.69			6.14	Cobalamin synthesis protein P47K
*groES*	*MSMEG_1582*	Rv3418c (99.0)	MAP4264 (96.0)	<0.0001	5.23			4.66	Cochaperonin GroES
*groEL*	*MSMEG_1583*	Rv3417c (81.1)	MAP4265 (82.5)	<0.0001	6.02			4.18	Molecular chaperone GroEL
—	*MSMEG_3787*	—	—	<0.0001	−4.00				d-Aminoacylase
*eda*	*MSMEG_3788*	—	—	<0.0001	−4.00				2-Dehydro-3-deoxyphosphogluconate aldolase
—	*MSMEG_3789*	—	—	<0.0001	−5.63				Inner membrane permease YgbN
*thpD*	*MSMEG_3898*	—	—	<0.0001	−4.94			−4.44	Ectoine hydroxylase
*ectC*	*MSMEG_3899*	—	—	<0.0001	−6.47			−4.55	l-Ectoine synthase
*ectB*	*MSMEG_3900*	—	—	<0.0001	−5.74			(−3.86)	Diaminobutyrate-2-oxoglutarate aminotransferase
*ectA*	*MSMEG_3901*	—	—	<0.0001	−4.05			(−2.82)	l-2,4-diaminobutyric acid acetyltransferase
—	*MSMEG_4509*	Rv2378c (75.4)	MAP2170c (74.4)	<0.0001	4.22				MbtG protein
*smtB*	*MSMEG_4486	Rv2358 (80.4)	MAP2138 (74.4)	<0.0001	94.76				ArsR family transcriptional regulator
—	MSMEG_4596	—	—	<0.0001	−116.50				Histidine kinase/Lpps protein
—	*MSMEG_5021*	—	—	<0.0001	−10.57			−6.08	Alcohol dehydrogenase
—	*MSMEG_5022*	—	—	<0.0001	−21.67			−5.91	Flavin-containing monooxygenase FMO
—	MSMEG_5180	—	MAP2636 (91.9)	<0.0001	−5.88				Hypothetical protein
—	*MSMEG_6062*	Rv1473 (42.5)	—	<0.0001	5.97			5.04	Fe uptake system permease
—	*MSMEG_6063*	—	—	<0.0001	8.73			8.69	Fe uptake system integral membrane protein
—	MSMEG_6239	—	—	<0.0001	−4.71				1,3-Propanediol dehydrogenase

a RCN, reference common name.

b —, no reference common name/orthologous gene.

c Italics indicate genes organized in an operon. Asterisks indicate the presence of one or more predicted Zur binding sites.

d *q* value of differentially expressed genes in MSMEGwt versus MSMEGΔ*zur* calculated by Rockhopper analysis. A *q* value of <0.01 is considered significant.

e Gene expression values of MSMEGΔ*zur* (Δ*zur*) and MSMEGΔ*smt*BΔ*zur* (ΔΔ) divided by gene expression values of MSMEGwt from RNA-Seq as well as results from transcriptome analysis after TPEN and ZnSO_4_ treatment. Values in parentheses indicate the differential expression of <4 genes which belong to the same operon.

f Putative function based on NCBI blastx analysis or the TB database.

In the group of zinc-dependent genes, which were all but four more highly expressed in the mutant than in the wild type, we found several extremely highly induced genes (>100- to 5,000-fold change). These included genes encoding ARPs (MSMEG_6065-6068 and MSMEG_6070), as well as two genes coding for CobW-like proteins (MSMEG_6048, MSMEG_6069) and a putative ABC transporter (MSMEG_6049-6051). In addition, a number of genes encoding other predicted cation transporters (MSMEG_6052, MSMEG_6058-6059), the predicted ZnuABC importer (MSMEG_6045-6047), the porin MspD (MSMEG_6057) as well as three genes of unknown function (MSMEG_6064, MSMEG_6071, MSMEG_6237) and one coding for a methyltransferase (MSMEG_6055) were classified as Zur and zinc dependent.

The majority of zinc-independent genes differentially expressed in MSMEGΔ*zur* showed only weak differences in expression compared to the wild type. However, we found some interesting clusters, e.g., one encoding a group of chaperones (MSMEG_0880, MSMEG_1582, MSMEG_1583) and one an iron uptake system (MSMEG_6062-6063), which were more highly expressed in the mutant, whereas gene clusters involved in the Entner-Doudoroff pathway (MSMEG_3787-3789), ectoine synthesis (expressed at higher salt concentrations) (MSMEG_3898-3901), and other metabolic pathways (MSMEG_0584, MSMEG_5021, MSMEG_5022, MSMEG_6239-6242) showed reduced expression compared to the wild type.

Two genes were highly affected by *zur* deletion but were zinc independent: expression of MSMEG_4486 (*smtB*) was ∼95-fold increased and transcription of MSMEG_4596, coding for a putative LppH protein, was strongly repressed (∼116-fold). The function of the latter gene is unknown.

The SmtB regulon was relatively small. Overall, only 12 genes were differentially expressed. Of these, only five belonged exclusively to the SmtB regulon (Fig. 3A; Table 5). Five genes were also present in the Zur regulon. Two genes were SmtB and zinc dependent (MSMEG_5117, MSMEG_0755). Only the expression of the cobalt-zinc-cadmium resistance gene MSMEG_0755 was affected by *smtB* deletion and TPEN and zinc treatment. While its expression was decreased (∼34-fold) upon zinc starvation and induced (∼14-fold) upon zinc excess, *smtB* deletion resulted in an ∼80-fold increase of expression. In the group of zinc-independent genes, we found two poorly characterized transcriptional regulator genes (MSMEG_3959, MSMEG_3960), *zur* (MSMEG_4487), one gene involved in metabolism (MSMEG_2659), and a gene encoding a protein of the ferritin family (MSMEG_6422). All these genes were more highly expressed in the mutant.

**TABLE 5:** Genes differentially expressed in MSMEG*ΔsmtB*, zinc dependent (including TPEN, zinc, and Δ*smtB*Δ*zur* results) or zinc independent

Gene category	RCN^*a*^	Annotation^*c*^	Orthologous gene (% similarity)		*q* value^*d*^	Fold change^*e*^				Putative function^*f*^
			MTB	MAP		Δ*smtB*	TPEN	Zn	ΔΔ	
Zinc dependent	—^*b*^	MSMEG_0755	Rv0359 (68.8)	MAP3865c (76.8)	<0.0001	80.92	−34.0	14.36	64.08	Cobalt-zinc-cadmium resistance protein
	—	MSMEG_5117	Rv1188 (68.3)	MAP2592c (71.6)	<0.0001	4.32		11.75	5.9	Proline dehydrogenase
	*rpmB*	*MSMEG_6068	Rv2058c (82.1)	—	<0.0001	7.17	449.65		4055.32	50S ribosomal protein L28
	—	MSMEG_6237	—	—	<0.0001	−5.39	7.40	−11.22	−4.62	Class I SAM-dependent methyltransferase
	—	*MSMEG_6240*	—	—	<0.0001	−4.64		(−3.01)	−8.48	Hypothetical protein
	—	*MSMEG_6241*	Rv2426c (45.9)	MAP2246c (44.9)	<0.0001	−5.14		(−3.74)	−8.66	ATPase AAA
	—	*MSMEG_6242*	—	—	<0.0001	−6.40		−4.64	−8.35	Alcohol dehydrogenase
Zinc independent	*ald*	MSMEG_2659	Rv2780 (80.6)	MAP2888 (76.2)	<0.0001	4.33				Alanine dehydrogenase
	—	MSMEG_3959	—	—	<0.0001	4.40			4.70	FadR-like transcriptional regulator
	—	MSMEG_3960	Rv0067c (45.8)	—	<0.0001	7.56			8.11	Transcriptional regulator
	*zur*	MSMEG_4487	Rv2359 (80.5)	MAP2139 (79.0)	<0.0001	14.13				Zinc uptake regulation protein
	—	MSMEG_6422	Rv3841 (71.8)	—	<0.0001	5.33				Ferritin family protein

a RCN, reference common name.

b —, no reference common name/orthologous gene.

c Italics indicate genes organized in an operon. Asterisks indicate the presence of one or more predicted Zur binding sites.

d *q* value of differentially expressed genes in MSMEGwt versus MSMEGΔ*smtB* calculated by Rockhopper analysis. A *q* value of <0.01 is considered significant.

e Gene expression values of MSMEGΔ*smtB* and MSMEGΔ*smtB*Δ*zur* (ΔΔ) divided by gene expression values of MSMEGwt from RNA-Seq as well as results from transcriptome analysis after TPEN and ZnSO_4_ treatment.

f Putative function based on NCBI blastx analysis or the TB database. Values in brackets () show differential expression <4 of genes, which belong to the same operon.

We then compared the Zur and SmtB regulons with the transcriptome of the MSMEG*ΔsmtBΔzur* double mutant. Among 81 differentially expressed genes in MSMEG*ΔsmtBΔzur* (see Table S3 in the supplemental material) with a >4-fold change, expression of 42 genes was congruent with genes of the single mutant regulons (Fig. 3B). Hence, expression of 79% of the genes regulated by Zur and SmtB (42 of 53 differentially expressed genes in total) was also affected in the double mutant. The 39 remaining genes could be assigned mostly to metabolism and stress response, possibly due to the metal imbalance, and are regulated by other regulators.

### Prediction and distribution of Zur and SmtB binding motifs in MSMEG.

To emphasize the direct involvement of SmtB and Zur in regulation, we were next interested in identification of putative binding motifs in the regulatory elements of regulated genes. We screened the MSMEG genome for putative SmtB and Zur binding sites by use of the MEME Suite and subsequent FIMO analysis. Due to the complex architecture of mycobacterial promoters, which can extend up to 2 kb (40), and the possible presence of more than one functional Zur box upstream of genes (41, 42), we considered motifs which were found in the 5′ untranslated region (UTR) up to −350 bp upstream of predicted open reading frames (ORFs) as putative binding sites. Of 46 motifs for Zur, 23 were found in the chosen 5′ UTR of ORFs (Table 6). Among those, we found 12 motifs located in the promoter-operator region of Zur-regulated genes (Table 6, indicated by boldface, and Tables 3 and 4, indicated by asterisks), i.e., the predicted ZnuABC transporter (MSMEG_6045-6047) and a second ABC transporter (MSMEG_6052), four CobW-like proteins (MSMEG_1122, MSMEG_1123, MSMEG_6048, MSMEG_6069), a methyltransferase (MSMEG_6055), and the set of genes encoding ARPs (MSMEG_6065-6068, MSMEG_6070). Some promoter-operators harbored more than one predicted motif (MSMEG_6047, MSMEG_6048, MSMEG_6055), which suggests a complex regulation of these genes, as already described for Streptomyces coelicolor and Cupriavidus metallidurans (41, 43). Notably, *smtB*-*zur* was also preceded by a putative Zur binding site. SmtB motif prediction by the MEME Suite based on the promoter sequences of the genes of the MSMEG SmtB regulon was not successful due to the low number of genes. Therefore, we used in our FIMO analysis the putative SmtB binding site of MTB, which was previously predicted from *in vitro* binding assays with *smtB*-*zur* promoter fragments (30). However, we were unable to confirm the presence of a SmtB binding site upstream of *smtB*-*zur* of MSMEG.

**TABLE 6:** Identification of Zur binding sites in MSMEG by FIMO analysis

RCN^*a*^	Annotation^*c*^	Orthologous gene (% similarity)		Putative function^*d*^	Position	Sequence comparison
		MTB	MAO			
						N C N T NNN GA N AA NNN TT NN C NNN A^*e*^
*rhaU*	MSMEG_0587	—	—	l-Rhamnose mutarotase/epimerase	−321	C C G T ACC GA A AA TGA TT GT C CGA A
—^*b*^	MSMEG_0883	—	MAP3940c (75.2)	Amidohydrolase	−145	C C T T GTC G TAG A ACG TT TT C GACT
*hemC*	MSMEG_0953	Rv0510 (81.6)	MAP4003 (82.2)	Porphobilinogen deaminase *hemC*	−38	C C T T GATA A C AA CCGA T CT C GAT A
—	***MSMEG_1123***	—	MAP1730c (43.0)	Cobalamin synthesis protein P47K (CobW)	−40	G C T T ACT GA A AA CTG TT CT C AAC A
—	***MSMEG_1122***	—	—	Cobalamin synthesis protein P47K	−2	GGT T GTT GA G AA CAG TT TT C AGT A
—	MSMEG_1288	—	—	Nuclease	−86	C C GAATT GA A AA CACC T GC C GAA A
—	MSMEG_3269	—	MAP1776c (44.0)	Putative sugar ABC transporter ATP-binding protein	−5	G C C T GAT G GC AA CAG TT TC C ATTG
—	MSMEG_3427	—	—	Metal-dependent hydrolase/beta-lactamase	−194	CGG T GAT GA A AA AGG T CTT C ATG A
—	MSMEG_4063	—	—	Amidohydrolase	−76	CAC T GTC GA AG A ACAC T TT C ATC A
—	MSMEG_4065	—	MAP2747 (38.0)	Fatty acid (feruloyl) CoA-synthetase	−33	CAG T ATC GA A AA CCAA T TC C GAC A
*glyS*	MSMEG_4485	Rv2357c (83.1)	MAP2137c (83.9)	Glycine-tRNA synthetase subunit beta	−139	T C AAATT G GA AA TCG TT TT C ATT A
ArsR	***MSMEG_4486***	Rv2358 (80.4)	MAP2138 (74.4)	ArsR family transcriptional regulator (SmtB)	−23	CGG T AAT GA A AA CGA TT TC C AATT
LpqT	MSMEG_5429	Rv1016c (57.1)	—	Hypothetical protein LpqT	−2	C C G T GAC GA TCGTCGC T GC C GTC A
MarR	MSMEG_5579	—	—	MarR family transcriptional regulator	−239	C C T T GCCC A AC A ACG TT TT C GAC A
—	***MSMEG_6047***	—	MAP3776c (57.0)	Cation ABC transporter substrate-binding protein	−47	AAG T AAT G CA AA CGG TT AT C GTT A
—	***MSMEG_6047***	—	—		−93	C C T T ATC GA A AA TGA TT TT C AAT A
—	***MSMEG_6048***	—	MAP3772c (72.6)	Cobalamin synthesis protein P47K (CobW)	−33	T C TTATT GA A AA TCA TT TT C GAT A
—	***MSMEG_6048***		—		−79	CGATAAC GA T AA CCG TT TG C ATT A
—	***MSMEG_6052***	—	MAP2210c (38.0)	ABC transporter ATP-binding protein	−34	CATTAAT GA T AA TCG TT TT C AAA A
—	***MSMEG_6055***	—	—	Class I SAM-dependent methyltransferase	−239	G C CTGTT GA A AA CCG TT AC C GATT
—	***MSMEG_6055***	—	—		−293	A C TTATT GA A AA TCG TT TT C GAC A
*rpmB*	***MSMEG_6068***	Rv2058 (82.1)	—	50S ribosomal protein L28	−58	G C T T ATT GA A AA CGA TT GT C ATT A
CobW	***MSMEG_6069***	Rv0106 (62.1)	MAP3770 (60.0)	CobW/P47K C domain protein	−57	CGA T AAT GA C AA TCG TT TT C AAT A

a RCN, reference common name.

b —, no reference common name/orthologous gene.

c Italics indicate genes organized in an operon. Boldface indicates genes found in the Zur regulon.

d Putative function based on NCBI blastx analysis or the TB database.

e Consensus sequence for Zur binding site obtained from the MEME Suite. Variable nucleotides are displayed as N, conserved nucleotides in FIMO-predicted binding sites are shaded in gray.

### *zitA* is regulated by SmtB.

**FIG 4:**
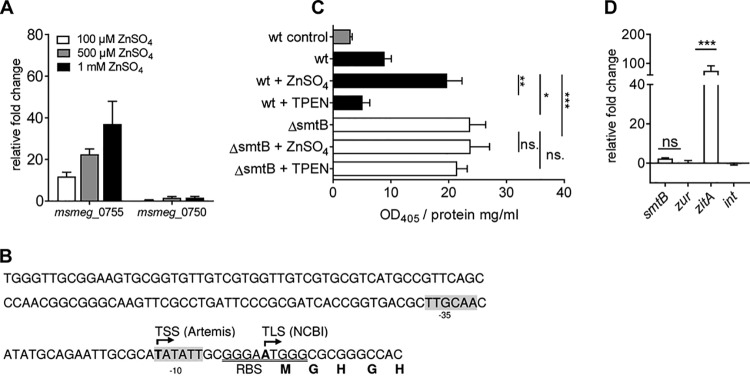
Analysis of SmtB-dependent *zitA* expression. (A) Gene expression of MSMEGwt grown in MB under standard conditions or increasing concentrations of ZnSO_4_ was analyzed by qRT-PCR with primers targeting MSMEG_0750 (primers 38/39) and MSMEG_0755 (primers 20/21). Shown are the results of three independent experiments (in duplicate), expressed as fold change (mean ± SEM) in comparison to the results for the untreated control and normalized to the housekeeping gene *gapdh*. (B) Schematic depiction of the *zitA* promoter region (NC_008596; positions 842101 to 842250). The putative transcription start site (TSS), determined by RNA-Seq and subsequent Artemis analysis, and the putative translation start site (TLS) according to NCBI are in bold. Putative −10 and −35 promoter sites, predicted by Neural Network Promoter Prediction (BDGP), are highlighted in gray, and the ribosome binding site (RBS) is underlined. (C) β-Galactosidase assay. MSMEGwt containing the vector only (wt control, gray bar), pJEM15-*zitA* (black bars), and MSMEGΔ*smtB* with pJEM15-*zitA* (white bars) were grown in MB and incubated with 100 μM ZnSO_4_ or 10 μM TPEN for 24 h or left untreated. β-Galactosidase activity in lysates was determined. Shown are the results (OD_405_/mg protein) of three independent experiments (mean ± SEM) in triplicate after 60 min of incubation. (D) ChIP assay. MSMEGΔ*smtB* complemented with pMV306-SmtB-Flag was cultivated in MB. ChIP from cross-linked lysates was performed using anti-Flag and anti-IgG2a (isotype control). DNA from ChIP and input control was subjected to qRT-PCR with primers targeting the *smtB* (MSMEG_4486) or *zitA* (MSMEG_0755) promoter or an intragenic region (*zur*, *int* primers 34 to 37). Shown are the results of at least three independent experiments in duplicate. Statistical analysis was performed using Student's *t* test (unpaired) with *P* values of <0.005 (**), <0.0005 (***), and <0.0001 (****).

FIMO analysis of the MSMEG genome did not predict any conclusive SmtB binding sites, neither for *smtB*-*zur* nor for any other SmtB-regulated gene, even though *smtB*-*zur* transcription repression by SmtB has been previously proposed for MTB and MSMEG (30). To dissect the relevance of SmtB in the regulation of genes in MSMEG, we analyzed MSMEG_0755, the *zitA* homologue of MSMEG, whose expression was strongly induced in the MSMEGΔ*smtB* mutant and which shows significant homologies to other CDF transporters (38). Since MSMEG_0750 has also been proposed as a gene homologue of *zitA* (34, 44), we first analyzed expression of both genes upon zinc stress. We observed a concentration-dependent induction of MSMEG_0755 but not of MSMEG_0750 (Fig. 4A), confirming the relevance of MSMEG_0755 in zinc homeostasis. In a next step, we fused the promoter of MSMEG_0755 (Fig. 4B) to *lacZ* using the expression vector pJEM15 and analyzed promoter activity by a β-galactosidase assay in MSMEGwt and MSMEGΔ*smtB* under standard conditions and upon zinc starvation (TPEN) or zinc excess (ZnSO_4_). These analyses revealed that promoter activity was significantly induced upon zinc stress in MSMEGwt and constantly high under all conditions in MSMEGΔ*smtB* (Fig. 4C). To analyze a direct involvement of SmtB in MSMEG_0755 regulation, we performed chromatin immunoprecipitation (ChIP) experiments. MSMEGΔ*smtB* was complemented with Flag-tagged SmtB, and DNA-protein complexes were precipitated after cross-linking with an antibody targeting the Flag tag or an isogenic IgG control antibody. DNA from ChIP was quantified by qRT-PCR with primers targeting the MSMEG_0755 promoter and additionally with primers targeting the promoter of *smtB*-*zur*, which was included as a SmtB but zinc independently regulated gene, as well as intragenic control primers, located in MSMEG_0755 and in the *zur* gene. As shown in Fig. 4D, ChIP led to the significant enrichment of MSMEG_0755 promoter DNA but not, however, of the *smtB*-*zur* promoter or the intragenic controls when grown under standard conditions. This indicated that SmtB binds to the MSMEG_0755 promoter despite the absence of a SmtB binding motif similar to MTB but suggests the presence of a different SmtB binding site, which has to be identified in future experiments.

### Regulation of *smtB*-*zur* expression is dependent on both regulators.

**FIG 5:**
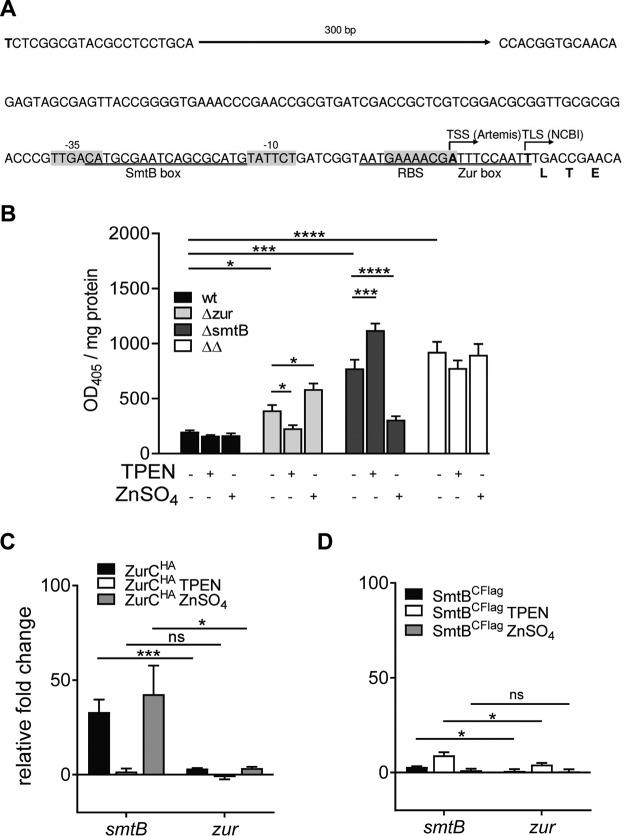
Analysis of *smtB*-*zur* regulation. (A) Schematic depiction of the *smtB*-*zur* promoter region (NC_008596; positions 4568740 to 4569210). The putative transcription start site (TSS), determined by RNA-Seq and subsequent Artemis analysis, and the putative translation start site (TLS) according to NCBI are in bold. Putative −10 and −35 promoter sites, predicted by BDGP, as well as a ribosome binding site (RBS) are highlighted in gray. Zur box (underlined) prediction was accomplished using the MEME Suite and subsequent FIMO analysis. SmtB box (underlined) was predicted by Canneva et al. (30). (B) β-Galactosidase assay. MSMEGwt (black bars), MSMEGΔ*zur* (light gray bars), MSMEGΔ*smtB* (dark gray bars), and MSMEGΔ*smtB*Δ*zur* (white bars) were transformed with pJEM15-*smtB1* harboring a 5′ UTR fragment as shown in panel A. Strains were grown in MB, incubated with 100 μM ZnSO_4_ or 10 μM TPEN for 24 h or left untreated, and lysed, and promoter activity was determined as described in the legend to Fig. 4. Shown are the results of three independent experiments (mean ± SEM) in triplicate after 21 min of incubation. Statistical analysis was performed by using one-way ANOVA with *P* values of <0.05 (*), <0.0005 (***), and <0.0001 (****). (C) ChIP assay. MSMEGΔ*zur* was complemented with pMV306-Zur-HA and treated with 10 μM TPEN (white bars) or 500 μM ZnSO_4_ (gray bars) for 2 h or left untreated (black bars). ChIP from cross-linked lysates was performed using anti-HA and anti-IgG1κ (isotype control). DNA from ChIP and input control was subjected to qRT-PCR with primers targeting the *smtB* promoter (MSMEG_4486) or an intragenic region (*zur*, MSMEG_4487). (D) SmtB-ChIP assay was performed as described for Zur but with MSMEGΔ*smtB* complemented with pMVM306-SmtB-Flag and precipitated with anti-Flag and anti-IgG2a. Shown are the results of at least three independent experiments in duplicate. Statistical analysis was performed using Student's *t* test (unpaired) with *P* values of <0.05 (*), <0.0005 (***), and <0.0001 (****).

**FIG 6:**
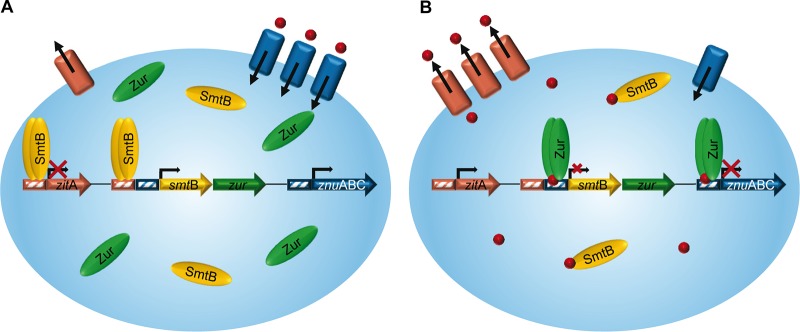
Model of SmtB-Zur regulation in MSMEG. (A) Under low zinc conditions, the SmtB homodimer binds to the DNA and represses expression of zinc exporter ZitA, whereas Zur detaches from the promoter of zinc importers such as *znuABC* and allows transcription and thus zinc uptake (red dots). Binding of SmtB to and release of Zur from the DNA also allows graded transcription of *smtB*-*zur.* (B) In the presence of zinc, the Zur homodimer binds to the DNA and represses *smtB*-*zur* and *znuABC* expression. In contrast, SmtB detaches from the DNA and *zitA* transcription is allowed, leading to zinc export.

Transcriptional analyses of the *smtB* and *zur* deletion mutants suggested that both SmtB and Zur regulate their own expression, since deletion of one regulator affected the expression of the other. This regulation was independent of zinc (Tables 1 and 2; Fig. S4). The data shown above, however, suggest that SmtB plays an inferior role in regulation, as no binding of SmtB to the promoter was visible under standard conditions (Fig. 4D). These findings were in contrast to those in other mycobacteria, as *smtB* expression was zinc dependent in MAP and regulated by SmtB in MTB (27, 29). Therefore, we studied the relevance of Zur and SmtB on MSMEG *smtB*-*zur* regulation in more detail. First, we analyzed promoter activity of the *smtB*-*zur* promoter after transformation of MSMEGwt, MSMEGΔ*zur*, MSMEGΔ*smtB*, and MSMEGΔ*smtB*Δ*zur* with pJEM15-*smtB1* (Fig. 5A). As shown in Fig. 5B, promoter activity was significantly enhanced in all mutants compared to that in the wild type. Upon TPEN or ZnSO_4_ treatment, no change in promoter activity was observed in the wild type or in the double mutant. In contrast, in MSMEGΔ*zur*, zinc depletion resulted in reduced promoter activity and zinc excess resulted in higher promoter activity. In MSMEGΔ*smtB*, promoter activity was lower upon ZnSO_4_ treatment and increased upon addition of TPEN. This indicated that in MSMEGΔ*zur*, the remaining regulator, SmtB, binds to the promoter when zinc is absent, whereas in MSMEGΔ*smtB*, Zur binds to the promoter when zinc is present (Fig. 6). Remarkably, zinc-dependent regulation was detectable only in the absence of the other regulator.

Unlike with MTB, a Zur binding site but no comprehensive SmtB binding motif could be identified by FIMO analysis in the *smtB* promoter of MSMEG. Thus, we performed ChIP analyses to elucidate the zinc-dependent direct binding of both regulators to their respective promoters, using MSMEGΔ*smtB* expressing Flag-tagged SmtB and MSMEGΔ*zur* complemented with hemagglutinin (HA)-tagged Zur. As shown in Fig. 5C, the amount of DNA of the *smtB* promoter was significantly enriched after Zur ChIP (anti-HA), whereas no enrichment was observed after SmtB ChIP (anti-Flag) and in the intragenic *zur* control (Fig. 5D), demonstrating direct binding of Zur to the promoter region of *smtB*-*zur* under standard growth conditions. Exposure of the bacteria to changing zinc concentrations influenced promoter binding of the regulators. Zur binding was significantly reduced upon zinc starvation (TPEN) but increased upon addition of zinc, indicated by the depletion or enrichment of DNA of the *smtB-zur* promoter region, respectively (Fig. 5C). Enrichment of promoter DNA after SmtB ChIP was visible only when zinc was depleted by TPEN (Fig. 5D). These data clearly show that in MSMEG, Zur and SmtB replace each other to regulate their own expression in response to zinc availability (Fig. 6).

## DISCUSSION

Despite the importance of zinc in mycobacterial metabolism, only a few studies have been published on mycobacterial zinc homeostasis. The importance of zinc was deduced mostly from studies focusing on mycobacterial pathogens (28, 29). All information concerning the transcriptional regulation of zinc homeostasis regulators SmtB and Zur has been obtained in MSMEG by heterologous expression of the MTB *smtB* and MSMEG *smtB* promoters (31).

Our transcriptomic analyses of the MSMEG response to zinc starvation and excess as well as the transcriptomes of MSMEGΔ*smtB* and MSMEGΔ*zur* mutants revealed a complete and complex picture of zinc-dependent gene regulation in MSMEG. Most genes in MSMEG affected by zinc encoded already known or putative zinc transport systems, as well as chaperones and alternative ribosomal proteins (ARPs). The known and common importer *znuABC* (MSMEG_6045-6047) was induced upon zinc starvation and Zur deletion in MSMEG. This finding was comparable to Zur-controlled, homologous *znuABC* in MTB (28) and MAP transporter MAP3776-3774c (29). Similar to other bacteria, MSMEG regulates expression of CobW-like proteins upon zinc starvation. Homologous genes in MAP and MTB have been shown to be regulated zinc dependently by Zur (28, 29). In Cupriavidus metallidurans, CobW proteins substitute for missing zinc importers (41). During zinc starvation, expression of genes *rpmB*, *rpmG*, *rpsN*, *rpsR*, and *rpmE2* encoding ARPs was highly induced and regulated by Zur. ARPs do not need zinc as a structural component and support MSMEG survival upon zinc limitation (45). Higher expression of such genes under limiting zinc conditions has also been described for MTB, MAP, Bacillus subtilis, Streptomyces coelicolor, and other bacteria (28, 29, 46, 47).

Concerning zinc export in MSMEG, our results revealed that expression of the zinc exporter MSMEG ZitA, encoded by MSMEG_0755, was directly regulated by SmtB, which is in accordance with findings on MTB ZitA (27). Thus, MSMEG seems to possess zinc storage and transport mechanisms similar to those of pathogenic mycobacteria. However, in contrast to pathogenic mycobacteria, the MSMEG zinc regulon comprises additional putative zinc uptake systems, such as the predicted Zn uptake transporter encoded by MSMEG_6049-6051 or *mspD* (MSMEG_6057). The Mycobacterium smegmatis porins (Msp) represent a group of four highly similar proteins (MspA to -D) specific for mycobacteria. MspA is involved in the uptake of glucose (48), phosphate (49), amino acid (50), and iron (51), and MspC is involved in copper uptake and growth (52). MspD differs from the other Msps in 18 amino acids only, but no substrate specificity has been described so far. Our study indicates that MspD may play a role in zinc uptake and might presumably provide an additional rapid channel-based exchange system for nutrients. Remarkably, MSMEG_6049, which is part of the MSMEG_6049-6051 operon, showed no homology to genes of pathogenic mycobacteria but to MVAN_5323 of the nonpathogenic Mycobacterium vanbaalenii. This gene is predicted to be part of a second Zur-regulated *znuABC* transporter. Due to the observed high expression in MSMEG treated with TPEN and in MSMEGΔ*zur*, the transporter operon MSMEG_6049-6051 might, therefore, encode an additional zinc importer in MSMEG. With regard to mycobacterial phylogeny, the absence of these transport systems in pathogenic mycobacteria suggests a better zinc availability in the host. In the concept of phylogenetic adaptation of pathogens to their host, this might have led to the loss of additional uptake systems.

Our analyses unraveled the transcriptional regulation of the MSMEG *smtB*-*zur* operon. The results indicate that in MSMEG, as with zinc import, regulation of zinc homeostasis also seems to be more complex than in pathogenic mycobacteria. SmtB and Zur are both sensitive measures of zinc concentration in other bacteria (20). Expression of the operon is autoregulated by SmtB in MTB (30) and/or is zinc dependent, as shown for MAP (29). Here, we found that in MSMEGwt, expression of *smtB* and *zur* was unaffected when zinc was either absent or in excess (Tables 1 and 2; see Fig. S4 in the supplemental material). This finding is in accordance with that in S. coelicolor, in which the amount of Zur protein under high or low zinc conditions remained constant (53). Strikingly, the apparently constitutive expression of *smtB*-*zur* in MSMEG was maintained by SmtB and Zur, depending on the availability of zinc. We found that both regulators affect the expression of their operon. Our promoter studies with the regulator deletion mutants revealed that both SmtB and Zur are needed for full regulatory control of their operon promoter. Furthermore, we showed that the regulation of *smtB*-*zur* expression is hierarchic. Zur seems to dominate the control and represses expression of the operon when zinc is freely available for the bacterium (Fig. 6B). We further show antagonistic binding of one regulator to the operator in the absence of the other (Fig. 5C and D). Hence, even in the case of imbalances in zinc homeostasis, expression of the operon is never uncontrolled. The importance of controlled *smtB*-*zur* expression is also indicated by our zone of inhibition (ZoI) assays (Fig. 2D to F). They show that differential gene expression in MSMEG lacking Zur or SmtB results in reduced zinc tolerance, which is not observable when both regulators are absent in the MSMEGΔ*smtB*Δ*zur* double mutant. Thus, the regulons of Zur and SmtB are necessary for maintaining MSMEG zinc homeostasis. If uncontrolled, differential gene expression alone cannot cope with zinc imbalances, which is illustrated by the reduced zinc tolerance of the MSMEGΔ*smtB*Δ*zur* double mutant when exposed to high zinc concentrations in the ZoI assays. However, the quality of zinc imbalances in the MSMEG single mutants needs to be clarified in future experiments. The antagonistic influence of Zur and SmtB on *smtB*-*zur* expression has not yet been reported for other bacteria. On first glance, such an autoregulation seems to be peculiar. However, a closer look reveals that it is necessary for a sensitive control of zinc homeostasis. In the case of sole regulation by Zur, the operon would be derepressed upon zinc limitation. This might lead to excessive synthesis of apo-Zur and may delay or impair the shutdown of zinc importers when zinc is sufficiently available. Moreover, additional SmtB produced simultaneously would impair its own deactivation and block zinc export to avoid zinc excess. On the other hand, sole regulation by SmtB would allow operon expression exclusively when MSMEG encounters an excess of zinc, or it would depend on the half-life of SmtB, since SmtB binds to the DNA in its apo form (Fig. 5D and 6A). This mechanism might be likely in organisms with stable zinc homeostasis. However, due to limited apo-Zur production, this mechanism would be disadvantageous for controlling zinc import after starvation. Therefore, the better capability to react quickly to changing zinc concentrations might also explain why Zur is the dominating regulator in this complex regulatory mechanism. The dominance of Zur also makes sense, as the environmental bioavailability of zinc is presumably low. Accordingly, in liquid culture, growth of MSMEG was hampered only in the absence of *zur* (Fig. 2B), which might be explained by an uncontrolled zinc influx due to the lack of counterregulation. This, however, needs to be proven by additional studies in the future.

In pathogenic MTB, *smtB*-*zur* regulation seems to be solely affected by SmtB (30). This suggests that mycobacteria in the host are exposed to more constant or higher zinc concentrations than they are in the environment, which would make regulation by SmtB more plausible and might reflect another example of mycobacterial adaptation to the host.

Overall, in the present study, we provide novel insights into the response of MSMEG to changing zinc concentrations. We show that MSMEG is well adapted to environmental changes in zinc availability. This is achieved by a sensitive regulation of the *smtB*-*zur* operon by both Zur and SmtB, allowing a balanced expression of the two regulators even under changing zinc concentrations and by the presence of additional putative zinc transporters in the genome. These mechanisms enable MSMEG to maintain reactivity and to constantly control intracellular zinc homeostasis.

## MATERIALS AND METHODS

### Bacterial strains, chemicals, and growth conditions.

Escherichia coli was grown in Luria-Bertani (LB) broth or LB agar supplemented with 50 μg/ml kanamycin, 100 μg/ml ampicillin, or 100 μg/ml hygromycin when necessary. Liquid cultures were incubated in a shaking incubator at 200 rpm/37°C. Competent cells were prepared as described earlier (54). E. coli strains DH5αF′ and 10-β were used for plasmid propagation.

Mycobacterium smegmatis mc^2^ 155 (MSMEG), mutants MSMEGΔ*smtB*, MSMEGΔ*zur* (previously constructed in our lab [29] and formerly designated MSMEGΔ*furB*), MSMEGΔ*smtB*Δ*zur*, and the complemented strains MSMEGΔ*smtB*^C^, MSMEGΔ*smtB*^CFlag^ MSMEGΔ*zur*^C^, MSMEGΔ*zur*^CHA^, and MSMEGΔ*smtB*^C^Δ*zur*^C^ were grown in Difco Middlebrook 7H9 medium (Becton Dickinson, Franklin Lakes, NJ, USA) supplemented with 10% OADC (0.06% oleic acid, 5% albumin, 2% dextrose, 0.085% NaCl, 0.003% catalase), 2.5% glycerol, and 0.025% tyloxapol (here referred to as MB) or on LB agar. Media were supplemented with kanamycin or hygromycin (both 50 μg/ml) if necessary. MSMEG competent cells were prepared as described by Parish and Stoker (55). Plasmids and strains used in this study are listed in Table S1, and primers are listed in Table S2, both in the supplemental material. All chemicals were purchased from Sigma-Aldrich (Munich, Germany) if not stated otherwise.

10.1128/mSystems.00880-19.5 TABLE S1 Bacterial strains and plasmids used in this study. Download Table S1, PDF file, 0.1 MB. Copyright © 2020 Goethe et al. 2020 Goethe et al. This content is distributed under the terms of the Creative Commons Attribution 4.0 International license.

10.1128/mSystems.00880-19.6 TABLE S2 Oligonucleotides used in this study. Download Table S2, PDF file, 0.1 MB. Copyright © 2020 Goethe et al. 2020 Goethe et al. This content is distributed under the terms of the Creative Commons Attribution 4.0 International license.

10.1128/mSystems.00880-19.7 TABLE S3 Genes differentially expressed in MSMEGΔ*smtB*Δ*zur*. Download Table S3, PDF file, 0.1 MB. Copyright © 2020 Goethe et al. 2020 Goethe et al. This content is distributed under the terms of the Creative Commons Attribution 4.0 International license.

### Growth experiments.

Cryostocks for zinc stress experiments and growth curves were prepared as follows: an overnight preculture of each strain was grown in MB to an optical density at 600 nm (OD_600_) of approximately 2.0 and inoculated into fresh medium to obtain an OD_600_ of 0.05. This culture was grown to an OD_600_ of 2.0. Bacteria were then harvested by centrifugation, resuspended in MB plus 10% glycerol, and intensively vortexed in the presence of glass beads, and remaining clumps were removed by low-speed centrifugation (300 × *g*). Aliquots of the cultures were frozen at – 80°C and stored until use. The number of CFU was determined prior to use.

Growth curves were obtained from bacteria grown in MB. Cultures were inoculated with bacteria from cryostocks to obtain an initial OD_600_ of 0.1 and incubated in a shaking incubator at 150 rpm, 37°C, for up to 48 h. Growth was monitored by measuring the OD_600_ every 3 h. To cover night times, we started one culture series in the morning and a second 12 h after the first. Four series of measurements in duplicate were conducted.

To determine zinc stress susceptibility, we performed zone of inhibition (ZoI) assays. Cryostocks were thawed on ice and diluted with MB, and 1 × 10^5^ cells were spread on 30-ml LB agar plates and allowed to dry for 30 min at 37°C. Filter discs 5 mm in diameter (Carl Roth, Karlsruhe, Germany) were applied under sterile conditions, and 10 μl of ZnSO_4_ solutions (concentrations of 25, 50, 75, and 100 mM) or sterile water (control) were applied to the filter disks. Agar plates were incubated for 3 days at 37°C. Inhibition zones were measured in millimeters. Experiments were repeated three times in triplicate.

### Preparation of nucleic acids, cDNA synthesis, and qRT-PCR.

Genomic DNA was extracted as described earlier (56). Plasmid DNA was prepared with a NucleoBond AX kit (Macherey and Nagel GmbH, Düren, Germany) according to the manufacturer’s protocol. Total RNA from standard and *N,N,N′,N′*-tetrakis (2-pyridylmethyl) ethylenediamine (TPEN)- or ZnSO_4_- treated MSMEGwt, mutants, and complemented strains was prepared using TRIzol as described by Eckelt et al. (29) or by using a Direct-zol RNA miniprep kit (Zymo Research, Bath, UK). cDNA was synthesized and analyzed by quantitative real-time PCR (qRT-PCR) as described by Eckelt et al. (29), using MSMEG_3084 (*gapdh*, primers 24/25) as a housekeeping gene.

### Construction and complementation of unmarked MSMEG mutants.

The generation of MSMEGΔ*zur* was described earlier (29). MSMEGΔ*smtB* and MSMEGΔ*smtB*Δ*zur* were constructed using the p2NIL/pGOAL19 system (57) (Addgene plasmids 20188 and 20190). Briefly, 1,500 bp upstream (A) and downstream (B) fragments flanking MSMEG_4486 (Δ*smtB*, primers 1 to 4) or the operon MSMEG_4486-87 (Δ*smtB*Δ*zur*, primers 1/2 and 5/6) were amplified from genomic DNA by standard PCR using Phusion or Q5 high-fidelity DNA polymerase (New England Biolabs, Beverly, MA, USA). Fragments were cloned into pJET1.2 (Thermo Fisher Scientific, Waltham, MA, USA) and sequenced. Plasmids containing the correct sequence were restriction digested with either HindIII/BsmBI (A) or BsmBI/KpnI (B) in the case of Δ*smtB* and with HindIII/BbsI (A) or BbsI/KpnI (B) in the case of Δ*smtB*Δ*zur*. The fragments were subsequently ligated to HindIII/KpnI-digested p2NIL, resulting in deletion plasmids p2NIL-MSMEG4486AB and p2NIL-MSMEG4486-87AB. A marker gene cassette from pGOAL19, digested with PacI, was then ligated into the deletion plasmids, designated p2NIL-MSMEG4486-Del and p2NIL-MSMEG4486-87-Del. MSMEGwt competent cells were electroporated with 1 to 5 μg of plasmid DNA which had been pretreated with 100 mJ UV light cm^−2^. Selection of mutants was performed as described by Parish and Stoker (57) with minor modifications as described earlier (29). Mutants were screened by PCR using primers 7 to 12.

Homologous complementation of MSMEGΔ*smtB* and MSMEGΔ*smtB*Δ*zur* was achieved by reintroducing *smtB* (MSMEG_4486) and *smtB*-*zur* (MSMEG_4486-87), respectively, both under the control of their own promoter, by PCR cloning from genomic DNA of MSMEGwt using primers 13/14 and 13/15, respectively. MSMEGΔ*zur* was complemented with *zur* (MSMEG_4487) under the control of the *smtB* promoter. For this, genomic DNA of MSMEGΔ*smtB* was used as the template for PCR with primers 13/14. The PCR fragments were digested with HindIII/XbaI and ligated to HindIII/XbaI-digested pMV306hyg. The resulting plasmids were designated pMV-MS*smtB*, pMV-MS*smtBzur* and pMV-MS*zur*, sequenced, and subsequently transformed into the corresponding mutant. Complemented strains were designated MSMEGΔ*smtB*^C^, MSMEGΔ*zur*^C^, and MSMEGΔ*smtB*^C^Δ*zur*^C^. In addition, for the ChIP assay, MSMEGΔ*smtB* and MSMEGΔ*zur* were complemented with a plasmid harboring a Flag-tagged *smtB* gene (pMV-MS*smtB*^Flag^) and an HA-tagged *zur* gene, respectively (pMV-MS*zur*^HA^). pMV-MS*smtB*^Flag^ was obtained by inverse PCR of pMV-MS*smtB* with primers 16/17 using Q5 polymerase (New England Biolabs, Beverly, MA, USA), pMV-MS*zur*^HA^ was constructed by ligation of AgeI/HindIII-digested pMV-MS*zur* and AgeI/HindIII-digested PCR product, which was generated with primers 18/19. After sequencing of the plasmid, successful Flag tag expression was confirmed via Western blot analysis (Fig. S1A and C) and functional complementation by qRT-PCR using primers 9 to 12 and 20 to 23, targeting *smtB*, *zur*, *zitA*, and MSMEG_6069 (Fig. S1B and D).

10.1128/mSystems.00880-19.1 FIG S1 Analysis of MSMEGΔs*mtB* complemented with Flag-tagged SmtB and MSMEGΔ*zur* with HA-tagged Zur. (A and C) Western blot analysis. MSMEGΔ*smtB* complemented with tagged (Δ*smtB*^CFlag^) or nontagged (Δ*smtB*^C^) SmtB and MSMEGΔ*zur* complemented with tagged (Δ*zur*^CHA^) or nontagged (Δ*zur*^C^) Zur were grown in MB. Fifty micrograms (MSMEGΔ*smtB*^CFlag^/MSMEGΔ*smtB*) or 100 μg (MSMEGΔ*zur*^CHA^/MSMEGΔ*zur*^C^) of extracted protein was run on a 12.5% polyacrylamide gel for approximately 3 h. Proteins were transferred to a polyvinyidene difluoride (PVDF) membrane (1 h, 12 V; semidry Bio-Rad Trans-Blot SD). The membrane was washed twice with TBST buffer (Tris-buffered saline with Tween 20), blocked for 1 h with 5% skim milk in TBST, washed, incubated overnight at 4°C with anti-Flag tag antibody diluted 1:500 in 5% skim milk (A) or with anti-HA tag antibody diluted 1:500 in 5% skim milk (C), washed, incubated for 1 h at room temperature with anti-rat IgG2a (A) or anti-mouse IgG1κ antibody (C), respectively, conjugated with alkaline phosphatase (1:10,000), washed, and finally incubated for 5 min with AP juice (PJK Biotech, Kleinbittersdorf, Germany). Signals were analyzed with an Integra chemoluminescence detector. (B and D) Analysis of functional complementation by qRT-PCR. MSMEGwt, MSMEGΔ*smtB*, MSMEGΔ*smtB*^C^, and MSMEGΔ*smtB*^CFlag^ were grown and lysed, RNA was extracted, and cDNA was analyzed by qRT-PCR for the expression of *smtB* (MSMEG_4486) and *zitA* (MSMEG_0755) or in the case of MSMEGΔ*zur*^CHA^ with primers targeting *zur* (MSMEG_4487) or MSMEG_6069. Results were normalized to *gapdh* and are presented as relative cDNA expression (2^−Δ^*^CT^*). Shown are the results of three independent replicates in duplicate. Download FIG S1, TIF file, 0.3 MB. Copyright © 2020 Goethe et al. 2020 Goethe et al. This content is distributed under the terms of the Creative Commons Attribution 4.0 International license.

10.1128/mSystems.00880-19.2 FIG S2 Operon structure of *zur* and *smtB* in different actinobacteria. A Geneious BLASTN search with the genome region covering *smtB*-*zur* of MSMEG mc^2^ 155 was performed with the whole-genome sequences of M. tuberculosis H37Ra, *M. thermoresistibile* NCTC 10409, Nocardia asteroides NCTC 11293, and Saccharothrix espanaensis DSM 44229. The sequence with the highest coverage in each genome was extracted, and a multiple alignment of the extracted sequences and MSMEG *smtB*-*zur* was performed with Geneious MAFFT. Annotations are as in the genome accessions. Download FIG S2, TIF file, 0.5 MB. Copyright © 2020 Goethe et al. 2020 Goethe et al. This content is distributed under the terms of the Creative Commons Attribution 4.0 International license.

10.1128/mSystems.00880-19.3 FIG S3 Characterization of different MSMEG deletion mutants. MSMEGwt (black bars), MSMEGΔ*zur*, MSMEGΔ*smtB*, and MSMEGΔ*smtB*Δ*zur* (ΔΔ) mutants (white bars) and the complemented strains MSMEGΔ*zur*^C^, MSMEGΔ*smtB*^C^, and MSMEGΔ*smtB*^C^Δ*zur*^C^ (gray bars) were grown in MB. Gene expression was analyzed by qRT-PCR. Shown are the relative expression levels of *smtB* (MSMEG_4486), *zur* (MSMEG_4487), and the adjacent gene MSMEG_4488 (primers 40/41) normalized to the housekeeping gene *gapdh*. Shown are the results from at least three independent replicates in duplicate, presented as relative cDNA expression (2^−Δ^*^CT^*). Download FIG S3, TIF file, 0.3 MB. Copyright © 2020 Goethe et al. 2020 Goethe et al. This content is distributed under the terms of the Creative Commons Attribution 4.0 International license.

10.1128/mSystems.00880-19.4 FIG S4 Zinc-independent regulation of MSMEG *zur* and *smtB*. MSMEGwt cultures were grown in MB and treated either with 10 μM TPEN or 100, 500, and 1,000 μM ZnSO_4_ for 2 h. Gene expression was analyzed by qRT-PCR. Shown are the results of three independent experiments (in duplicate), expressed as fold change (mean ± SEM) compared to the untreated control and normalized to the housekeeping gene *gapdh*. Download FIG S4, TIF file, 0.1 MB. Copyright © 2020 Goethe et al. 2020 Goethe et al. This content is distributed under the terms of the Creative Commons Attribution 4.0 International license.

### RNA deep sequencing and analysis.

Total RNA for transcriptome analysis was prepared from cultures in MB. MSMEG strains were grown in MB to an OD_600_ of approximately 2.0. MSMEGwt cultures for zinc starvation or excess were divided in two and subsequently treated with 10 μM TPEN or 100, 500, and 1,000 μM ZnSO_4_ or were left untreated (control) for 2 h. RNA deep sequencing of MSMEGwt treated with TPEN or zinc and MSMEG mutants was performed as described before (29). The quality and integrity value RNA integrity number (RIN) >8 of total RNA was controlled on an Agilent Technologies 2100 bioanalyzer or checked by a Qubit 2.0 fluorometer. The RNA sequencing libraries were generated from 250 ng total RNA using a Ribo-Zero rRNA removal kit (bacteria) (Illumina, San Diego, CA) for rRNA depletion, followed by a ScriptSeq v2 RNA-Seq library preparation kit (Epicentre, WI, USA) according to the manufacturers’ protocols. Briefly, 50-bp single-end sequencing was performed on a HiSeq 2500 or a NovaSeq 6000 PE50 (Illumina, San Diego, CA) with a mean output of 15 × 10^6^ reads per sample. BWA v. 0.7.5 was applied for the alignment of sequences against the reference strain MSMEG mc^2^ 155 (NCBI accession no. NC_008596) with an average mapping rate of 90% and a share of rRNAs around 6%. SAMtools were used for storing nucleotide sequence alignments. Further data analysis was performed with the Rockhopper tool (58). Genes with a false discovery rate (*q* value) of <0.01 were considered significantly differentially expressed. Exceptions were some genes in the Zur regulon which could not be detected as differentially expressed by the Rockhopper tool but were checked individually by qRT-PCR and/or by the Integrative Genomics Viewer tool (59) and indicated by a *q* value of 1.0 in Table 3.

### β-Galactosidase assay.

The 5′ untranslated region (5′ UTR) of MSMEG_4486 (*smtB*) (471-bp fragment; NC_008596 positions 4568740 to 4569210) and MSMEG_0755 (*zitA*) (150-bp fragment; NC_008596 positions 842101 to 842250) were amplified with primers 26 to 29 from genomic MSMEGwt DNA with Q5 high-fidelity polymerase (New England Biolabs, MA, USA). PCR fragments were ScaI/BamHI digested and ligated into ScaI/BamHI-digested pJEM15, resulting in plasmids pJEM15-*smtB1* and pJEM15-*zitA*. Construction was controlled by PCR, restriction enzyme digestion, and sequencing. Plasmids were subsequently transformed into MSMEGwt, MSMEGΔ*smtB*, MSMEGΔ*zur*, or MSMEGΔ*smtB*Δ*zur*, grown in MB overnight (150 rpm, 37°C) to an OD_600_ of approximately 2.0, and treated with 10 μM TPEN or 100 μM ZnSO_4_ for 24 h or left untreated (control). Protein extraction, determination of protein concentration, and β-galactosidase assay were conducted as previously described (29). Absorption was measured at 405 nm in a fluorescence reader (Genios Pro; Tecan, Männedorf, Switzerland) at 37°C for 60 min at 3-min intervals to determine the linear enzyme activity. Promoter activity was calculated as fluorescence at 405 nm/protein mg/ml.

### ChIP and ChIP–qRT-PCR.

Strains MSMEGΔ*smtB*^CFlag^ and MSMEGΔ*zur*^CHA^ were grown in MB to an OD_600_ of approximately 2.0 as described before and treated with 10 μM TPEN or 100 μM ZnSO_4_ for 2 h or left untreated (control). Cultures were then cross-linked with 1% methanol-free formaldehyde for 20 min at room temperature on a rolling incubator. The reaction was quenched with 0.125 M glycine, cells were harvested and washed twice with 50 mM Tris-HCl plus 10 mM EDTA (pH 7.5), and lysates were prepared as described by Eckelt et al. (29) with minor modifications. Cells were resuspended in Tris-HCl plus EDTA (pH 7.5) buffer and lysed in a ribolyzer, and DNA was sheared by sonification (ultrasonic intensity duty cycle constant) for 20 min. Cell debris was removed by centrifugation (11,000 × *g*, 30 min, 4°C). Protein concentration was determined by MicroBCA as described previously (29). Chromatin immunoprecipitation (ChIP) was performed as follows: 250 μg protein from lysates was mixed with 30 μl salmon sperm DNA–protein A/G agarose matrix (Santa Cruz Biotechnology, TX, USA) and 2 μg anti-DYKDDDK (Flag) antibody or IgG2a rat antibody (unspecific binding control) for SmtB-Flag ChIP and 2 μg anti-HA antibody or IgG1κ mouse antibody for Zur-HA ChIP (all antibodies from BioLegend, CA, USA), and samples were adjusted with ChIP DLP buffer {16.7 mM Tris-HCl, 1.2 mM, 167 mM NaCl, 1.1% Triton X-100, 0.01% SDS, 1 mM AEBSF [4-(2-aminoethyl)benzenesulfonyl fluoride hydrochloride], pH 8.0} to a final total volume of 800 μl and incubated overnight at 4°C (rotating incubator). Protein A/G-antibody-DNA complexes were pelleted, and 100 μl of the supernatant from each sample was retained as input control. Beads were washed as described by Braunstein et al. (60) and finally resuspended in 55 μl 10 mM Tris-HCl–1 mM EDTA–1% SDS buffer (pH 8.0). DNA-protein complexes were eluted from agarose beads by heating (1,200 rpm, 30 min, 70°C) and centrifugation (11,000 × *g*, 30 s, 4°C). The supernatant was transferred to a new tube and diluted with 150 μl TE buffer (10 mM Tris-HCl, 1 mM EDTA, pH 8.0). DNA from ChIP and input samples was isolated upon treatment with RNase (250 μg/ml) for 30 min at 37°C, proteinase K (1 μg/ml) for 2 h at 37°C, and 160 mM NaCl at 65°C overnight, followed by standard phenol-chloroform-isoamyl alcohol (25:24:1) extraction and precipitation in the presence of 40 μg glycogen with 140 mM LiCl and ethanol 99.8% for 30 min at room temperature. DNA was pelleted, washed with 80% ethanol, and dissolved in distilled water. DNA from ChIP was analyzed by qRT-PCR in a total volume of 20 μl containing 2 μl of ChIP-DNA, 10 μl SYBR green mix (Qiagen), and 200 nM specific primers by using a Stratagene MX3005P cycler. Primer pairs were selected for either the *smtB* and *zitA* promoter (primers 30 to 33) or intragenic controls (primers 34 to 37). For primer efficacy, 1 μg of each input DNA was pooled, serially diluted, and used for every single primer pair (61).

The PCR conditions were 95°C for 20 min, 95°C for 45 s, 58°C for 1 min, and 72°C for 1 min, followed by a melting curve of the product as the control. Data were analyzed as follows: in a first step, threshold cycle (*C_T_*) values of the input were subtracted from *C_T_* values of antibody-treated samples (Δ*C_T_*). Δ*C_T_* values of isotype controls (IgG2a, IgG1κ) were then subtracted from Δ*C_T_* values of anti-Flag- or anti-HA-precipitated samples (ΔΔ*C_T_*) and calculated as log_2_ to determine the relative fold change. For statistical analyses, the relative fold change of promoter primers (*smtB*, *zitA*) was related to relative fold change of control primers (*zur*, *zitAint*).

### Bioinformatics and statistics.

Differentially expressed genes obtained by Rockhopper analysis were further analyzed with NCBI blastx and the TB database (http://genome.tbdb.org) to identify putative protein functions. Putative Zur binding sites in MSMEG (accession no. NC_008596) were identified by FIMO analysis. Published MTB Zur binding sites of Rv0106, Rv2069, *rpmB2*, *rpmB1*, Rv3017c, and Rv3019c (28), as well as the Zur box of MAP3736c (29) were used to generate the consensus sequence of a Zur box ([C/G]C[T/C/G]T[A/G][T/A][T/C]GA[A/T]AA[T/C][A/C/G][A/G]TT[T/G][T/C]C[A/G][T/A][T/C]A) by MEME Suite.

Single nucleotides in sequences are conserved, and nucleotides in brackets are variable. In Table 6, variable nucleotides are displayed as N. This sequence was subsequently submitted to FIMO analysis (62). The genomic locations of all detected binding sites were determined and considered putative Zur binding sites (Zur box) within the range −350 up to +5 nucleotides relative to predicted translation start sites (TLS). Promoter prediction was accomplished using Neural Network Promoter Prediction (BDGP) (https://www.fruitfly.org/seq_tools/promoter.html). Statistical analyses were performed with GraphPad Prism 8.0.1 for Windows (GraphPad Software, San Diego, CA, USA). Determination of predicted transcriptional start sites was conducted with Artemis software (63). Identification of bacteria with adjacent *smtB* and *zur* genes was performed using NCBI BLASTN (https://blast.ncbi.nlm.nih.gov/Blast.cgi?PROGRAM=blastn&PAGE_TYPE=BlastSearch&LINK_LOC=blasthome) with the nucleotide sequence *smtB*-*zur* of MSMEG (NC_008596), bases 4569200 to 4569951. BLASTN was set as follows: database nucleotide collection (nr/nt), exclude models (XM/XP) and uncultured/environmental sample sequences. Hits were filtered (query cover, 85 to 100%; identity, 75 to 100%), and one strain of each species was selected for further analysis, with the exception of M. avium, for which the two subspecies M. avium subsp. *hominissuis* and M. avium subsp. *paratuberculosis* were used. A “distance tree of results” was generated in NCBI (tree method, neighbor joining; maximum sequence difference, 0.75), and the homologous sequences were downloaded. These were used for a multiple MAFFT alignment in Geneious. If a strain exhibited two or more homologous sequences of different sizes, the longest homologous sequence was kept for tree building with the Geneious tree builder (genetic distance model, Jukes Cantor; tree building method, unweighted pair group method using average linkages [UPGMA]). Venn diagrams were created with InteractiVenn (http://www.interactivenn.net) (64), and a heat map was created using normalized expression values of data obtained from Rockhopper analysis after transcriptome sequencing (RNA-Seq), presented as log_2_, and plotted with Microsoft Office Professional Plus 2016 Excel (version 16.0.4966.1000).

### Data availability.

Data from RNA-Seq are available as FASTA files at the European Nucleotide Archive under accession number PRJEB35513.
