# Supplementary material for: Critical Role of Zur and SmtB in Zinc Homeostasis of Mycobacterium smegmatis
Source: mSystems. 2020 Apr 21;5(2):e00880-19. doi: 10.1128/mSystems.00880-19 (PMC7174638; doi:10.1128/mSystems.00880-19)
Supplement: TABLE S1 [file mSystems.00880-19-st001.pdf]

Table S1: Bacterial strains and plasmids used in this study

Bacterial strains

Escherichia coli DH5αF'	F'/endA1 hsdR17 (rκ- mκ- supE44 thi-1 recA1 gyrA (Nalr relA1 Δ(lacZYA-argF)U169 deoR [ϕ80dlacΔ(lacZ)M15]	Raleigh et al., 1989
M. smegmatis mc^{2}155	Transformable strain of the isolate M.smegmatis ATCC 607	Snapper et al., 1990
M. smegmatis Δ*zur* (MSMEGΔ*zur*)	markerless *zur* (*msmeg* 4487) deletion mutant of *M. smegmatis* mc^{2}155	Eckelt et al., 2014
M. smegmatis Δ*smt* B (MSMEGΔ*smt* B)	markerless *smt* B (*msmeg* 4486) deletion mutant of *M. smegmatis* mc^{2}155	this work
M. smegmatis Δ*smt* BΔ*zur* (MSMEGΔ*smt* BΔ*zur*)	markerless *smt* B-*zur* (*msmeg* 4486) doublemutant of *M. smegmatis* mc^{2}155	this work
MSMEGΔ*zur*^{C}	MSMEGΔ*zur* complemented with pMV306hyg containing *msmeg* 4487 under control of the *msmeg* 4486 promotor	this work
MSMEGΔ*smt* B^C	MSMEGΔ*smt* B complemented with pMV306hyg containing *msmeg* 4486 under control of its own promotor	this work
MSMEGΔ*smt* BΔ*zur*^{C}	MSMEGΔ*smt* B-*zur* complemented with pMV306hyg containing *msmeg* 4486-4487 under control of the *msmeg* 4486 promotor	this work
MSMEGΔ*smt* B^{CFlag}	MSMEGΔ*smt* B complemented with pMV306hyg containing FLAG-tagged *msmeg* 4486 under control of its own promotor	this work
MSMEGΔ*zur*^{CHA}	MSMEGΔ*zur* complemented with pMV306hyg containing HA-tagged *msmeg* 4487 under control of the *msmeg* 4486 promotor	this work

Plasmids

pJET^{TM}1.2	subcloning blunt end suicide vector, *amp*	ThermoFisher Scientific, Waltham, MA, USA
p2NIL	*E. coli* - mycobacterium shuttle vector for gene manipulation, *kan*	Parish & Stoker 2000
pGOAL19	*hyg*, P_{AG85}-*lac* Z, P_{hsp60}-*sac* B, *Pac* I cassette vector, *amp*	Parish & Stoker 2000
pMV306hyg	integrative *E.coli* - mycobacterium shuttle vector, *hyg*	Stover et al. 1991
pMV-MSsmtB	pMV306hyg containing *msmeg* 4486 under its own promotor	this work
pMV-MSsmtB^{Flag}	pMV306hyg containing FLAG-tagged *msmeg* 4486 under its own promotor	this work
pMV-MSzur	pMV306hyg containing *msmeg* 4487 under control of the *msmeg* 4486 promotor	this work
pMV-MSzur^{HA}	pMV306hyg containing HA-tagged *msmeg* 4487 under control of the *msmeg* 4486 promotor	this work
pMV-MSsmtBzur	pMV306hyg containing *msmeg* 4486 and *msmeg* 4487 under its own promotor	this work
p2NIL_MSMEG4486_AB	p2NIL containing 1500 bp up- and downstream fragments of *msmeg* 4486	this work
p2NIL_MSMEG4486_Del	p2NIL containing *Pac* I restricted selection marker gene cassette of pGOAL19 and 1500 bp up- and downstream fragments of *msmeg* 4486	this work
p2NIL_MSMEG4486-87_AB	p2NIL containing 1500 bp up- and downstream fragments of *msmeg* 4486-87	this work
p2NIL_MSMEG4486-87_Del	p2NIL containing *Pac* I restricted selection marker gene cassette of pGOAL19 and 1500 bp up- and downstream fragments of *msmeg* 4486-87	this work
pJEM15	promoterless *E. coli* - mycobacterium shuttle vector for *lac* Z-fusion, *kan*	Timm et al. 1994
pJEM-MSSmtB1	pJEM15 containing 471 bp 5'UTR of *msmeg* 4486 (pos. 4568740-4569210) [a]	this work
pJEM-MS0755-1	pJEM15 containing 150 bp 5'UTR of *msmeg* 0755 (pos. 842101-842250) [a]	this work

[a] The position numbers given related to GenBank accession no. NC_008596

Raleigh FA, Lech K, Brent R 1989: Selected topics from classical bacterial genetics. In Current protocols in molecular biology. Edited by Ausubel FM. New York, USA: Publishing Associates and Wiley Interscience:1.4.1-1.4.14.
Snapper SB, Melton RE, Mustafa S, Kieser T, Jacobs WR Jr. 1990. Isolation and characterization of efficient plasmid transformation mutants of Mycobacterium smegmatis. Mol.Microbiol. 4.11 : 1911-19.
Eckelt E, Jarek M, Fromke C, Meens J, Goethe R. 2014. Identification of a lineage specific zinc responsive genomic island in Mycobacterium avium ssp. paratuberculosis. BMC Genomics 15:1076.
Parish T, Stoker NG. 2000. Use of a flexible cassette method to generate a double unmarked Mycobacterium tuberculosis tlyA plcABC mutant by gene replacement. Microbiology 146 (Pt 8):1969-1975.
Stover CK, de lC, V, Fuerst TR, Burlein JE, Benson LA, Bennett LT 1991: New use of BCG for recombinant vaccines. Nature, 351: 456-460.
Timm J, Lim EM, Gicquel B 1994: Escherichia coli-mycobacteria shuttle vectors for operon and gene fusions to lacZ: the pJEM. J Bacteriol, 176:6749–6753.
