# Supplementary material for: Critical Role of Zur and SmtB in Zinc Homeostasis of Mycobacterium smegmatis
Source: mSystems. 2020 Apr 21;5(2):e00880-19. doi: 10.1128/mSystems.00880-19 (PMC7174638; doi:10.1128/mSystems.00880-19)
Supplement: TABLE S2 [file mSystems.00880-19-st002.pdf]

Table S2: Oligonucleotides used in this study

No.	Oligonucleotides	Description or sequence (5' to 3')	Source/ reference
1	oMSMEG_4486-A fw	GATAAGCTTACACCTTGTCGATCGCGATG<br>(forward primer situated at position 4567714-4567733)[a], *Hind* III restriction site underlined	this work
2	oMSMEG_4486-A rev	GATCGTCTCGTGGTCGTGGGCCTCG<br>(reverse primer situated at position 4569214-4569229)[a], *Bsm* BI restriction site underlined	this work
3	oMSMEG_4486-B fw	ATCGTCTCGACCACGCCAGCGAGGACCG<br>(forward primer situated at position 4569525-4569537)[a], *Bsm* BI restriction site underlined	this work
4	oMSMEG_4486-B rev	CTGGTACCCACCTGCACCGCGCCGACAT<br>(reverse primer situated at position 4571019-4571038)[a], *Kpn* I restriction site underlined	this work
5	oMSMEG_4486-4487B_fw	GATCGTCTCGACCAATCAGGTGAGGAAC<br>(forward primer situated at position 4569952-4569965)[a], *Bsm* BI restriction site underlined	this work
6	oMSMEG_4487B_rev	CATGGTACCGGCACAAGATGGGTGAGGCGGTGCTCATC<br>(reverse primer situated at position 4571423-4571451)[a], *Kpn* I restriction site underlined	Eckelt et al., 2014
7	oMSMEG_4486-4487-Del_fw	CGTAGATCTCGCCGGATTGG<br>(forward primer situated at position 4568989-4569008)[a]	this work
8	oMSMEG_4487-Del_rev	GTGAGGTCGTCGAGGAGTTG<br>(reverse primer situated at position 4570202-4570221)[a]	this work
9	oRTMSMEG-furB fw	CACCGGTGAGTCGGTGTA<br>(forward primer situated at position 4569735-4569752)[a]	this work
10	oRTMSMEG-furB rev	CTCGATCGTGTGGCTGAC<br>(reverse primer situated at position 4569880-4569897)[a]	this work
11	oRTmsmeg4486 fw	ATCGCGATCGTTTTGCAG<br>(forward primer situated at position 4569317-4569334)[a]	this work
12	oRTmsmeg4486 rev	GAGCCGGTAGAGCACCTC<br>(reverse primer situated at position 4569461-4569478)[a]	this work
13	oMS4487-K1	GATCTCTAGAGTAGCGAGTTACCGGGGTGAAAC<br>(forward primer situated at position 4569076-4569098)[a], *Xba* I restriction site underlined	this work
14	oMS4487-K2	GATCAAGCTTATCATCACGTCTGGTTCCCGGCGGGCCG<br>(reverse primer situated at position 4569928-44569951)[a], *Hind* III restriction site underlined	this work
15	oMS4487-K5	GATCAAGCTTATCATCACCTGCGGTCCTCGCTGGCGTG<br>(reverse primer situated at position 4569521-4569544)[a], *Hind* III restriction site underlined	this work
16	oSmtB-FlagOL_fw	**AGACGATGACGACAA**GTGATGATAAGCTTATCGATGTCGACGTA<br>(forward primer situated at position 4569172-4569208)[a], bases of FLAG-tag bold, bases of pMV plasmid underlined	this work
17	oSmtB-FlagOL_rev	**CATCGTCTTTGTAGTC**CCTGCGGTCCTCGCTGGCGTGCGCGATC<br>(reverse primer situated at position 4569514-4569541)[a], bases of FLAG-tag bold	this work
18	oFurB-CFlag-HA_fw	GATCCAGCTGGTAGCGAGTTACCGGGGTGAAACCCG<br>(forward primer situated at position 4569076-4569101)[a], *Pvu* II restriction site underlined	this work
19	oFurB-CHA_rev	GATCAAGCTTTCA**AGCGTAATCTGGAACATCGTATGGGTA**CGTCTGGTTCCCGGCGGGCC<br>(reverse primer situated at position 4569929-4569948)[a], *Hind* III restriction site underlined, bases of HA-tag bold	this work
20	oRTmsmeg_0755fw	GGCGCCTATATGGAGGTC<br>(forward primer situated at position 841645-841662)[a]	this work
21	oRTmsmeg_0755rev	GTGACGATTCGGACAGGAT<br>(reverse primer situated at position 841476-841494)[a]	this work
22	oRTmsmeg_6069fw	AGTACGCATCGTCGAGTTCA<br>(forward primer situated at position 6134480-6134499)[a]	this work
23	oRTmsmeg_6069rev	GCTGATTCGATCCACATGAC<br>(reverse primer situated at position 6134610-6134629)[a]	this work
24	oRT_MSMEG_3084fw	GTGCCAAGAAGGTCATCATC<br>(forward primer situated at position 3157638-3157657)[a]	Eckelt et al., 2014
25	oRT_MSMEG_3084rev	ACTCGTCATTGAGCACCTTG<br>(reverse primer situated at position 3157780-3157799)[a]	Eckelt et al., 2014
26	oJEM-MSSmtB1_fw	GATCAGTACTTCTCGGCGTACGCCTCCT<br>(forward primer situated at position 4568740-4568757)[a],*Sca* I restriction site underlined	this work
27	oJEM-MSSmtB1_rev	CATGGATCCTGTTCGGTCAAATTGGAAA<br>(reverse primer situated at position 4569192-4569210)[a], *BamH* I restriction site underlined	this work
28	oJEM-MS0755-1_fw	GATCAGTACTTGGGTTGCGGAAGTGCGG<br>(forward primer situated at position 842250-842233)[a],*Sca* I restriction site underlined	this work
29	oJEM-MS0755-1_rev	CATGGATCCGTGGCCCGCGCCCATTCCC<br>(reverse primer situated at position 842101-842115)[a], *BamH* I restriction site underlined	this work
30	oCHIP-MSsmtB_fw	GGTGCAACAGAGTAGCGAGT<br>(forward primer situated at position 4569065-4569084)[a]	this work
31	oCHIP-MSsmtB_rev	GTGTTCGGTCAAATTGGAAA<br>(reverse primer situated at position 4569192-4569211)[a]	this work
32	oCHIP-MSZitA_fw	GTGTGGTGACTGTGATCGTG<br>(forward primer situated at position 842084-842103)[a]	this work
33	oCHIP-MSZitA_rev	GTGGTTGTCGTGCGTCAT<br>(reverse primer situated at position 842208-842225)[a]	this work
34	oCHIP-MSzur_fw	CACCATCACCACCACCTG<br>(forward primer situated at position 4569769-4569786)[a]	this work
35	oCHIP-MSzur_rev	CTCGATCGTGTGGCTGAC<br>(reverse primer situated at position 4569880-4569867)[a]	this work
36	oCHIP-MSZitAint_fw	GTGGTGACGGTGACGATG<br>(forward primer situated at position 841586-841603)[a]	this work
37	oCHIP-MSZitAint_rev	GGCGCCTATATGGAGGTC<br>(reverse primer situated at position 841645-841662)[a]	this work
38	oRTmsmeg0750_fw	CTCGTCGTCTTCATCGAGTC<br>(forward primer situated at position 838645-838664)[a]	this work
39	oRTmsmeg0750_rev	CCGATGATGTAACCCACCT<br>(reverse primer situated at position 838471-838489)[a]	this work
40	oRTmsmeg4488 fw	ACGCCATCTCCTCGATCT<br>(forward primer situated at position 4570051-4570068)[a]	this work
41	oRTmsmeg4488 rev	TGCACCGTGAGGTCGT<br>(reverse primer situated at position 4570212-4570227)[a]	this work

[a] The position numbers given related to GenBank accession no. NC_008596

Eckelt E, Jarek M, Fromke C, Meens J, Goethe R. 2014. Identification of a lineage specific zinc responsive genomic island in Mycobacterium avium ssp. paratuberculosis. BMC Genomics 15:1076.
