# Supplementary material for: Critical Role of Zur and SmtB in Zinc Homeostasis of Mycobacterium smegmatis
Source: mSystems. 2020 Apr 21;5(2):e00880-19. doi: 10.1128/mSystems.00880-19 (PMC7174638; doi:10.1128/mSystems.00880-19)
Supplement: TABLE S3 [file mSystems.00880-19-st003.pdf]

Table S3: Genes differentially expressed in MSMEGΔ*smt*BΔ*zur*

RCN[a]	Annotation	Orthologue genes (% similarity) MAP	Mtb	q-value	Fold change ΔΔ	ΔsmtB	Δzur	Putative function[d]
-	MSMEG_0217			< 0.0001	4.92			alcohol dehydrogenase
-	MSMEG_0302			< 0.0001	4.06			peptidase. S9A/B/C families
-	MSMEG_0424			< 0.0001	4.56			Hsp20/alpha crystallin family protein
-	MSMEG_0755	MAP3865c (76.8)	Rv0359 (68.8)	< 0.0001	64.08	80.92		cobalt-zinc-cadmium resistance protein
groEL	MSMEG_0880	MAP3936 (95.0)	Rv0440 (93.4)	< 0.0001	6.62		7.94	molecular chaperone GroEL
-	MSMEG_1122	-	-	< 0.0001	6.14		4.69	hypothetical protein
-	MSMEG_1123	MAP1730c (43.1)	-	< 0.0001	7.5		8.64	cobalamin synthesis protein
-	MSMEG_1236			< 0.0001	4.09			Mpr protein
-	MSMEG_1392			< 0.0001	6.79			alcohol dehydrogenase
groES	MSMEG_1582	MAP4264 (96.0)	Rv3418c (99.0)	< 0.0001	4.66		5.23	co-chaperonin GroES
groEL	MSMEG_1583	MAP4265 (82.5)	Rv3417c (81.1)	< 0.0001	4.18		6.02	molecular chaperone GroEL
aspA	MSMEG_1677			< 0.0001	-4.01			aspartate ammonia-lyase
mmsA	MSMEG_2449			< 0.0001	9.04			methylmalonate-semialdehyde dehydrogenase
-	MSMEG_2450			< 0.0001	5.31			hypothetical protein
-	MSMEG_3297			< 0.0001	-5.07			CadC family transcriptional regulator
-	MSMEG_3300			< 0.0001	-7.0			oxidoreductase. Gfo/Idh/MocA family protein
-	MSMEG_3301			< 0.0001	-4.36			hypothetical protein
thpD	MSMEG_3898	-	-	< 0.0001	4.44		-4.94	ectoine hydroxylase
ectC	MSMEG_3899	-	-	< 0.0001	4.55		-6.47	L-ectoine synthase
	MSMEG_3900	-	-	< 0.0001	(-3.86)		-5.74	diaminobutyrate--2-oxoglutarate aminotransferase
	MSMEG_3901	-	-	< 0.0001	(-2.82)		-4.05	L-2.4-diaminobutyric acid acetyltransferase
-	MSMEG_3946			< 0.0001	4.16			transmembrane protein
-	MSMEG_3948			< 0.0001	4.05			acyltransferase
-	MSMEG_3959	-	-	< 0.0001	4.7	4.40		transcriptional regulator
-	MSMEG_3960	-	Rv0067c (45.8)	< 0.0001	8.11	7.56		transcriptional regulator
-	MSMEG_4207			< 0.0001	-43.0			universal stress protein family protein
-	MSMEG_4208			< 0.0001	-45.33			integral membrane protein
-	MSMEG_4209			< 0.0001	-91.0			integral membrane protein
-	MSMEG_4210			< 0.0001	-57.75			hypothetical protein
-	MSMEG_4486			< 0.0001	-13.5		94.76	ArsR family transcriptional regulator
cysNC	MSMEG_4978			< 0.0001	-4.43			bifunctional sulfate adenylyltransferase subunit 1/adenylylsulfate kinase
cysD	MSMEG_4979			< 0.0001	-4.29			sulfate adenylyltransferase subunit 2
-	MSMEG_5021	-	-	< 0.0001	-6.08		-10.57	alcohol dehydrogenase
-	MSMEG_5022	-	-	< 0.0001	-5.91		-21.67	flavin-containing monooxygenase FMO
-	MSMEG_5117	MAP2592c (71.6)	Rv1188 (68.3)	< 0.0001	5.9	4.32		proline dehydrogenase
-	MSMEG_5241			< 0.0001	4.29			GAF family protein
-	MSMEG_5244			< 0.0001	4.19			DevR family transcriptional regulator
-	MSMEG_5576			< 0.0001	5.67			D-mannonate oxidoreductase
-	MSMEG_5906			< 0.0001	4.18			acyl-CoA dehydrogenase
-	MSMEG_5907			< 0.0001	4.50			acyl-CoA dehydrogenase
-	MSMEG_5908			< 0.0001	6.33			acyl-CoA synthetase
-	MSMEG_5909			< 0.0001	6.22			oxidoreductase
-	MSMEG_5913			< 0.0001	4.77			dioxygenase
-	MSMEG_5922			< 0.0001	7.45			lipid-transfer protein
-	MSMEG_5923			< 0.0001	5.0			acetyl-CoA acetyltransferase
-	MSMEG_5992			< 0.0001	5.66			hypothetical protein
-	MSMEG_5993			< 0.0001	5.82			acyl-CoA dehydrogenase
-	MSMEG_5994			< 0.0001	6.43			acyl-CoA dehydrogenase
-	MSMEG_5995			< 0.0001	4.89			P450 heme-thiolate protein
-	MSMEG_5997			< 0.0001	4.44			hypothetical protein
-	MSMEG_5998			< 0.0001	4.21			hypothetical protein
-	MSMEG_6037			< 0.0001	4.01			2-hydroxy-6-ketonona-2.4-dienedioic acid hydrolase
-	MSMEG_6045	MAP3774c (70.1)	Rv2060 (43.7)	< 0.0001	24.42		24.62	heavy metal ABC transporter inner membrane protein
-	MSMEG_6046	MAP3775c (61.2)	-	< 0.0001	19.9		20.65	ABC transporter ATP-binding protein
-	MSMEG_6047	MAP3776c (56.4)	-	< 0.0001	25.5		26.24	cation ABC transporter periplasmic cation-binding protein
-	MSMEG_6048	MAP3772c (72.6)	-	10.000	2,903.0		3,288.5	cobalamin synthesis protein/P47K
-	MSMEG_6049	-	-	< 0.0001	443.56		494.44	hypothetical protein
-	MSMEG_6050	-	-	< 0.0001	849.0		917.25	solute-binding lipoprotein
-	MSMEG_6051	-	-	< 0.0001	632.0		777.0	pseudo ABC transporter transmembrane protein
-	MSMEG_6052	-	-	< 0.0001	23.42		18.17	ABC transporter ATP-binding protein
bluB	MSMEG_6053			< 0.0001	19.9			cob(II)yrinic acid a.c-diamide reductase
-	MSMEG_6055	-	-	10.000	25.5		498	hypothetical protein
-	MSMEG_6057	-	-	< 0.0001	393		435	MspD protein
-	MSMEG_6058	MAP3384 (77.7)	Rv3270 (77.2)	< 0.0001	9.59		9.21	cadmium transporting P-type ATPase
-	MSMEG_6059	MAP3383 (69.1)	Rv3269 (71.6)	< 0.0001	11.73		13.42	hypothetical protein
-	MSMEG_6062	-	Rv1473 (42.5)	< 0.0001	5.04		5.97	Fe uptake system permease
-	MSMEG_6063	-	-	< 0.0001	8.69		8.73	Fe uptake system integral membrane protein
-	MSMEG_6064	-	-	< 0.0001	21.76		27.42	lipoprotein
rpsR	MSMEG_6065	MAP3767c (63.9)	Rv2055c (63.4)	10.000	3,080.0		5,107.67	30S ribosomal protein S18
rpsN	MSMEG_6066	MAP3768c (75.2)	Rv2056c (72.3)	10.000	3,983.5		5,778.5	30S ribosomal protein S14
rpmG	MSMEG_6067	MAP3769c (92.6)	Rv2057c (87.0)	10.000	5,015.0		5,856.67	50S ribosomal protein L33
rpmB	MSMEG_6068	-	Rv2058c (82.1)	10.000	4,055.32	7.17	5,372.69	50S ribosomal protein L28
-	MSMEG_6069	MAP3770 (57.8)	Rv0106 (62.1)	10.000	4,340.0		3,492.0	CobW/P47K domain-containing protein
rpmE2	MSMEG_6070	MAP3771 (79.3)	-	10.000	1,603.0		1,460.33	50S ribosomal protein L31
-	MSMEG_6071	MAP0485c (66.9)	Rv3577 (68.3)	< 0.0001	33.42		27.95	metallo-beta-lactamase superfamily protein
-	MSMEG_6072			< 0.0001	5.0			citrate transporter
-	MSMEG_6237	-	-	< 0.0001	-4.62	-5.39	-6.93	hypothetical protein
-	MSMEG_6240	-	-	< 0.0001	-8.48	-4.64	-24.38	hypothetical protein
-	MSMEG_6241	MAP2246c (44.9)	Rv2426c (45.9)	< 0.0001	-8.66	-5.14	-24.63	ATPase AAA
-	MSMEG_6242	-	-	< 0.0001	-8.35	-6.40	-27.59	alcohol dehydrogenase
-	MSMEG_6272			< 0.0001	12.44			NAD-glutamate dehydrogenase

[a]Reference Common Name.

[d]Putative function based on NCBI blastx analysis or tbdb.org database.
