# Supplementary figures and images for: Critical Role of Zur and SmtB in Zinc Homeostasis of Mycobacterium smegmatis
Source: mSystems. 2020 Apr 21;5(2):e00880-19. doi: 10.1128/mSystems.00880-19 (PMC7174638; doi:10.1128/mSystems.00880-19)

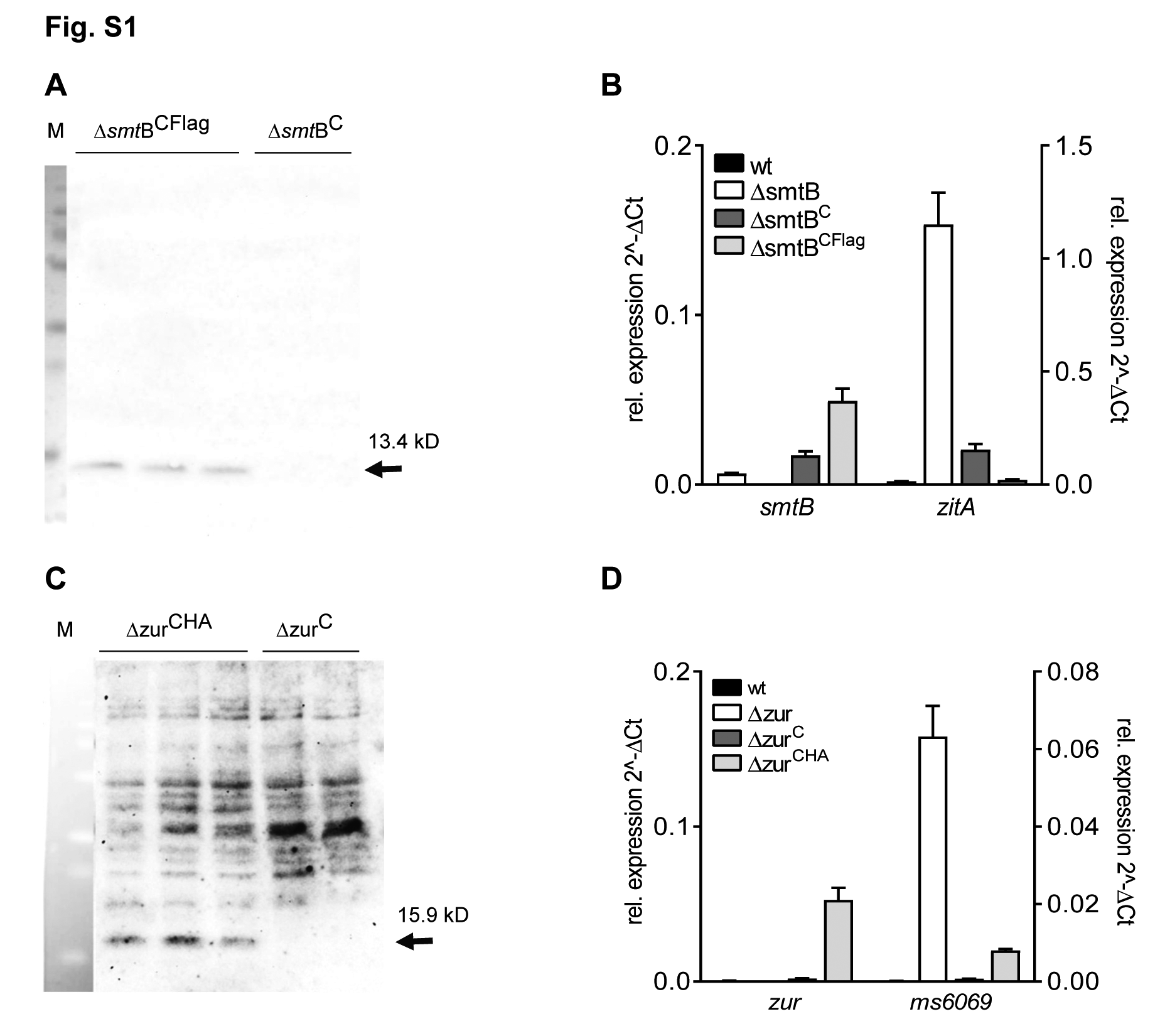

Supplement: FIG S1 [file mSystems.00880-19-sf001.tif]

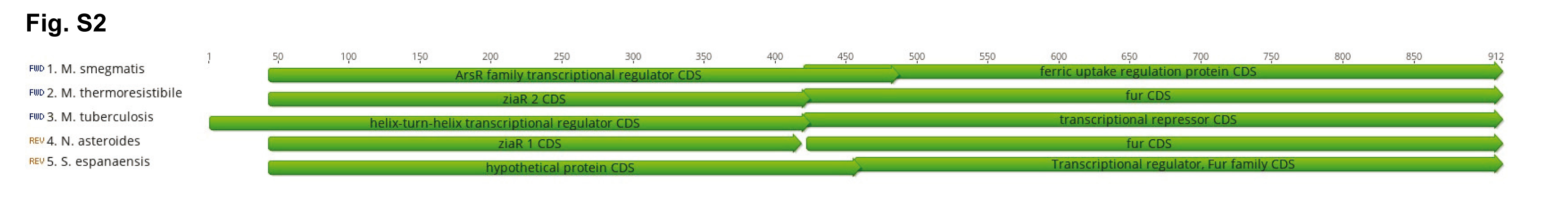

Supplement: FIG S2 [file mSystems.00880-19-sf002.tif]

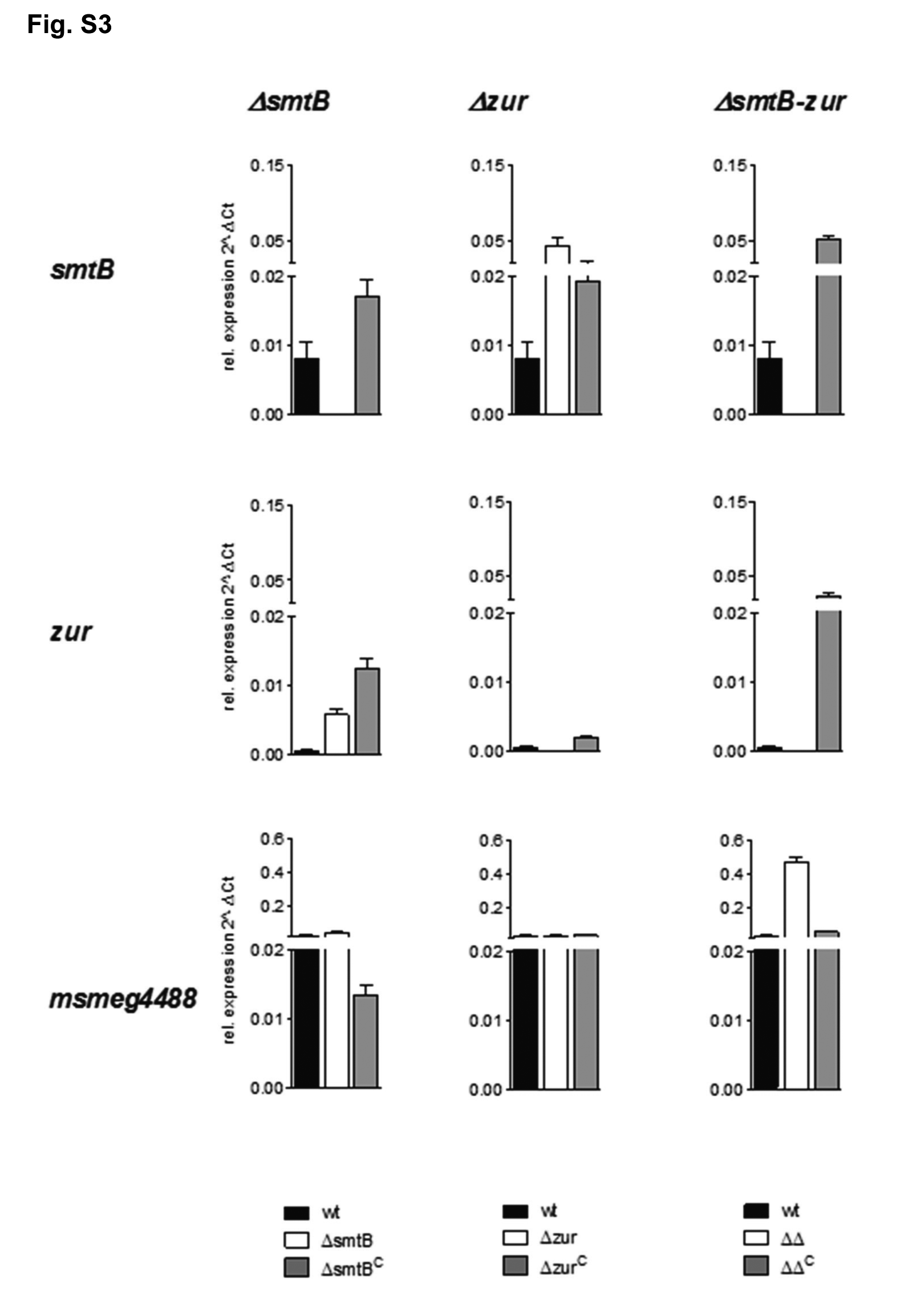

Supplement: FIG S3 [file mSystems.00880-19-sf003.tif]

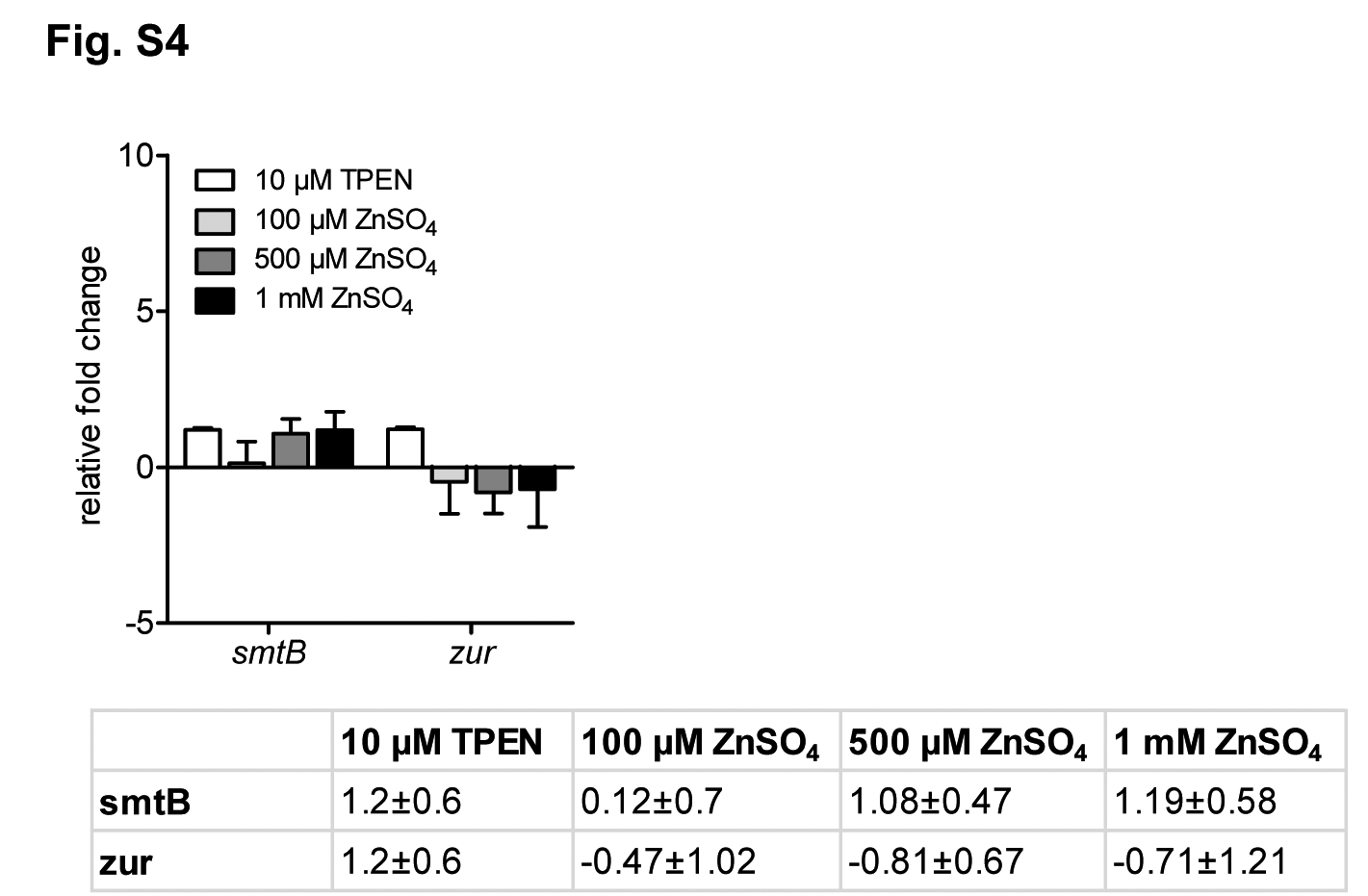

Supplement: FIG S4 [file mSystems.00880-19-sf004.tif]
